# Assessment of the ecological integrity and fish community structures of the uMngeni River, KwaZulu-Natal, South Africa

**DOI:** 10.2989/16085914.2025.2564685

**Published:** 2025-11-18

**Authors:** Pumla Dlamini, Colleen T Downs, Matthew Burnett, Gordon O’Brien

**Affiliations:** 1 Centre for Functional Biodiversity, School of Life Sciences, University of KwaZulu-Natal, Pietermaritzburg, South Africa; 2 Gulbali Institute, Charles Sturt University, Albury, New South Wales, Australia

**Keywords:** anthropogenic, ecological status, FRAI, multivariate analysis, water resource management

## Abstract

The uMngeni River is economically important as it provides water to two of the largest cities in KwaZulu-Natal Province, South Africa. As such, protecting the river and the life within it is also of great importance. In this study, we used fish community structure as an indicator of ecosystem health by assessing how fish communities responded to changes in habitat composition and water quality as a consequence of anthropogenic activities, using the Fish Response Assessment Index (FRAI). We used multivariate statistical analyses to determine differences in fish communities and drivers of change in these communities. We found that the ecological integrity of the uMngeni River (and its tributaries) tended to degrade from upper to lower reaches in response to various anthropogenic activities. Examples of degradation included flow modification and migration barriers from instream structures, the introduction of invasive fish species, and water quality alterations from rural and urban settlements. Multivariate analyses showed that variation among the sites selected in this study was significantly driven by changes in velocity-depth classes, substrate type, and water quality, all of which can be influenced by flow modifications. The FRAI ecological scores and multivariate analyses presented in the study provide a baseline for managing the uMngeni River’s fish communities, highlighting that flow regime, river fragmentation, and alien invasive species negatively impact these.

## Introduction

Freshwater makes up just 2.5% of all water on earth, and of that, less than 1% is available for human use, as most freshwater is contained in polar ice caps (Geise 2010). Most of the freshwater available for human use comes from rivers, which also provide a plethora of other ecosystem services on which they rely (Costanza et al. 1997; Yeakley et al. 2016). River anthropogenic services include water purification, transportation, power generation, food supply, and water supply (for domestic, agricultural and industrial use) (Tejerina-Garro et al. 2005; Yeakley et al. 2016). Unfortunately, riverine ecosystems are among the most intensively affected by anthropogenic activities (Tejerina-Garro et al. 2005; Dudgeon 2014). In South Africa, particularly, increased urbanisation and industrialisation have caused increasing deterioration of the water quality of most river systems (Sibanda et al. 2015), the effects of which have been exacerbated by water storage reservoirs and human use (Ashton 2007; Ashton 2010; Wepener and Chapman 2012; Fouchy et al. 2018).

The pressures and demands of a growing economy and human population, such as South Africa’s, have great impacts on riverine habitat structure, natural flow regimes, water quality and fish diversity (Saunders et al. 2002; Vidal 2008; Fouchy et al. 2018; O’Brien et al. 2019). Introducing invasive species also detrimentally affects the ecosystem health and biodiversity of its rivers (Vander Zanden 1999; Rahel 2000; Gao et al. 2019). This can sometimes result in drastic reductions of certain species (Ashton 2010). The severity of the impact of anthropogenic activities on rivers makes the assessment of the state and health of these ecosystems of utmost importance. There are various ways to assess ecosystem health, including environmental components (abiotic) and the use of different biological organisms (biotic) at various levels of biological organisation (Richardson et al. 2011; Fouchy et al. 2018). The levels of biological organisation that can be used range from the molecular level to the community level (Wepener 2008; Richardson et al. 2011; Wepener and Chapman 2012).

The ability to sustain a balanced biotic community is one of the best indicators of a healthy aquatic ecosystem (Karr 1981; O’Brien et al. 2014). Fish communities are considered extremely useful when assessing the biotic integrity of an aquatic ecosystem as they are relatively easy to identify, include an array of species (representing a range of trophic levels), are sensitive to water quality and are usually present in even the smallest streams (Karr 1981; O’Brien et al. 2019; Evans et al. 2022). Additionally, fish generally are good environmental indicators as they are widely distributed and mobile in aquatic systems, and have a relatively long lifespan (DWAF 1999; Gamito et al. 2012; O’Brien et al. 2011; Levin et al. 2019). There are, however, some disadvantages associated with using fish as indicators of ecological integrity, such as the high tolerance of some species to environmental change (including habitat degradation) and pollution, and the biased nature of sampling methods (Whitfield and Elliott 2002; Harrison and Whitfield 2004; Cabral et al. 2012; Gamito et al. 2012; Collins et al. 2017). However, the benefit of using fish as indicators outweighs these disadvantages (Avenant et al. 2010; Gamito et al. 2012; Evans et al. 2022).

Fish communities can be defined or classified in numerous ways depending on the study’s aims, the characteristics of the fish community that the study focuses on and the type of quantitative analysis used (Jackson et al. 2001, 2016; Dempsey 2019). One of the methods used to classify fish communities (and other species communities) is multivariate statistical approaches (O’Brien et al. 2009; Wepener et al. 2011; Evans et al. 2022). Multivariate statistics not only summarise and predict community patterns, but they also provide an objective approach to identifying fish assemblages and the impact that environmental conditions have on them (Taylor et al. 1993; Magnuson et al. 1998; Jackson et al. 2001; Wepener et al. 2011).

Another approach to studying riverine fish communities is to use the Fish Response Assessment Index (FRAI) (Kleynhans 1999). The FRAI is used to routinely determine the well-being of fish communities in southern African freshwater ecosystems by assessing the response of fish communities to changes in environmental conditions (Kleynhans 2007; Malherbe et al. 2016; Levin et al. 2019; Evans et al. 2022). The environmental conditions normally include water quality and habitat integrity (Kleynhans 2007; Levin et al. 2019; Evans et al. 2022). The uMngeni River, KwaZulu-Natal, South Africa, is one of the most socio-economically important rivers in the region but is under severe anthropogenic pressure (WRC 2002; Ramulifho 2015; Namugize et al. 2018).

Our study aimed to assess the ecological integrity of fish communities in the uMngeni River using FRAI and multivariate statistical techniques. We predicted that the uMngeni River would generally be in a poor state, largely because of the anthropogenic activities in and around the river. In this study, we evaluated the present ecological integrity and fish community structures of the uMngeni River using multiple lines of evidence, including community metric measures (FRAI) and multivariate statistical analyses of differences in fish communities and drivers of changes in these communities.

## Materials and Methods

### Study area

**Figure 1:**
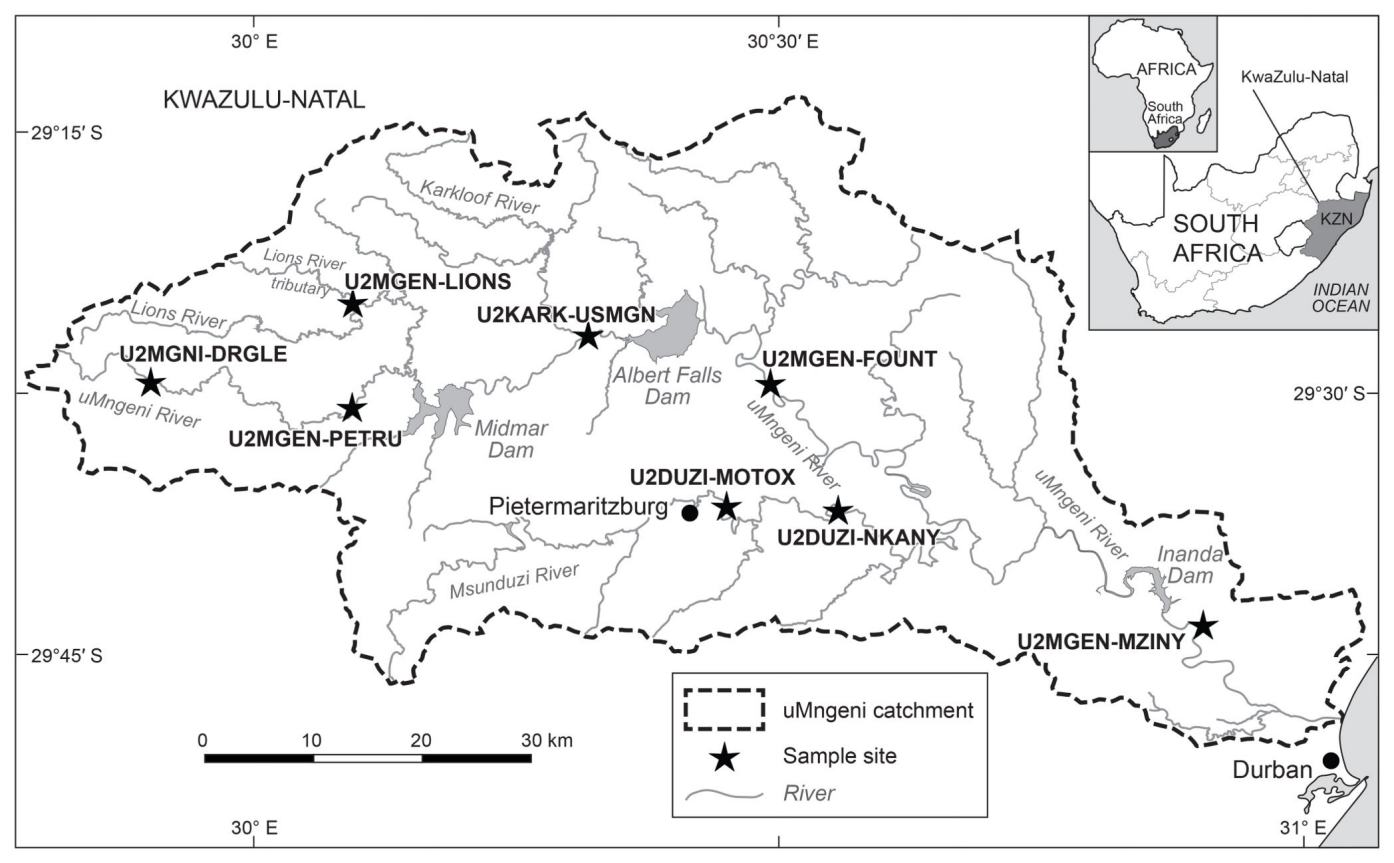
Map of sampling sites on the uMngeni River, KwaZulu-Natal Province, South Africa, in the present study. Site coordinates are presented in the Supplementary Table S1

The uMngeni catchment in KwaZulu-Natal Province, South Africa, has an area of 4 440 km^2^ with a mean annual rainfall of 921 mm (Umgeni Water 2016; Hughes et al. 2018). It is a summer rainfall region, ranging from an alpine-type climate in and along the Drakensberg Mountains to a more temperate summer rain climate in the Midlands region and a subtropical perennial rainfall area along the coast (Umgeni Water 2016). Mean annual ambient temperatures range between 14 and 22°C, and the catchment is generally characterised by grassland, with areas of thicket and bushland and forest patches, with a high portion of the catchment modified for agricultural activities (Umgeni Water 2016; Hughes et al. 2018). The study area comprised of eight sites in the uMngeni catchment and included sites on the uMngeni (including tributaries) and Msunduzi rivers (Figure 1). The sites’ selection coincides with sites used in the national River Eco-status Monitoring Programme (REMP).

#### Upper uMngeni River near Dargle (U2MGNI-DRGLE)

Situated 30 km upstream of Midmar Dam, at the Umgenipoort Research Centre, this sampling site was in the Dargle Conservancy on the main stem of the uMngeni River in the KwaZulu-Natal Midlands (Figure 1, Supplementary Figure S1a). The area surrounding this site was comprised mainly of pastoral grasslands and some forests (including indigenous forests and exotic timber plantations). The trees lining the riverbank at this site included the invasive black wattle (*Acacia mearnsii*), which were later felled during the study. The site was downstream of two wetlands, which are considered the source of the uMngeni River.

#### Upstream of Midmar Dam (U2MGEN-PETRU)

The sampling site U2MGEN-PETRU was in the Howick area (Figure 1, Supplementary Figure S1b). The site was situated in an agricultural area and was directly (about 50 m) below a weir. Agricultural activities sometimes negatively impacted this site (authors, pers. obs.).

#### The Lions River tributary (U2MGEN-LIONS)

The sampling site U2MGEN-LIONS was in Caversham Valley, near Howick (Figure 1, Supplementary Figure S1c). The site was surrounded by agriculture, exotic timber plantations and a few scattered residential areas (including Caversham Mill Restaurant). A waterfall and arch bridge were less than 300 m upstream of the site.

#### The Karkloof River tributary (U2KARK-USMGN)

The U2KARK-USMGN site was located at the base of the Karkloof River, before it joins the uMngeni River (Figure 1, Supplementary Figure S1d). There were numerous bridge crossings and a weir about 3.5 km upstream of this site. The site was surrounded by agricultural land, exotic timber plantations, and savannah (authors, pers. obs.).

#### The uMngeni River mid-catchment (U2MGEN-FOUNT)

This sampling site was in Fountainhill Estate in the Midlands, outside Wartburg (Figure 1, Supplementary Figure S1e). Fountainhill Estate forms part of a conservancy (Central Umgeni Conservancy), and the site was mostly surrounded by a mix of bush and grassland with agricultural activity in the surrounding area. The site is ~20 km below Albert Falls Dam and, at times, was negatively impacted by flow regulation (authors, pers. obs.).

#### The Msunduzi River, Pietermaritzburg City (U2DUZI-MOTOX)

Sampling site U2DUZI-MOTOX was on the Msunduzi River, which flows through the urban area of Pietermaritzburg City and contained generic anthropogenic waste (authors, pers. obs.; Figure 1, Supplementary Figure S1f). The site was ~2 km below the city’s Wastewater Treatment Works and ~36 km downstream of Henley Dam. There were some agricultural (sugarcane) and peri-urban activities in the surrounding areas. The site was negatively impacted by pollution from the urban and peri-urban land cover and flow modifications from Henley Dam.

#### The Msunduzi River lower catchment (U2DUZI-NKANY)

Sampling site U2DUZI-NKANY was also on the Msunduzi River in the rural area of Nkanyezini (Figure 1, Supplementary Figure S1g). The site was often used by the local community for sand mining and watering their cattle. There was also the invasive water hyacinth *Eichhornia crassipes* present at this site. This site was generally impacted negatively by sand mining, cattle presence and water hyacinth invasion while sampling (authors, pers. obs.).

#### The uMngeni River lower catchment (U2MGEN-MZINY)

Sampling site U2MGEN-MZINY was located within the township of iNanda (Figure 1, Supplementary Figure S1h). The site was ~5 km below the iNanda Dam wall. The area surrounding the site was predominantly township settlements or peri-urban with some forest or bushveld patches. This site was negatively impacted by modified flow caused by the dam and runoff from the surrounding human settlements (authors, pers. obs.).

### Field sampling

We sampled fish communities from all eight riverine sites (also used in the REMP) in the uMngeni and Msunduzi Rivers, KwaZulu-Natal (Figure 1). We collected fish using electro-fishing techniques and active and passive netting techniques (Barbour et al. 1999; Rodtka et al. 2015). Netting techniques included the use of a 6 m long, 1.5 m deep seine net with a bag that we pulled through both shallow (< 1 m) and deep (> 1 m) habitats. We sampled the habitats according to established REMP velocity-depth categories, including slow (< 0.3 m/s), deep (> 0.5 m) and shallow (> 0.5 m), fast (> 0.3 m/s), deep (> 0.3 m) and shallow (< 0.3 m) (James and King 2010). All available substrate and cover features were sampled, including marginal and aquatic vegetation, undercut banks and root wads. We identified fish using Skelton (2001), measured their standard length (SL), and then returned them to the river alive. We collected fish samples three times at each site, with high flow from 10–15 May 2017, low flow from 11–18 August and 7 September 2017, and a high flow from 7–10 November 2017.

### Water physico-chemical characteristics (water quality)

We measured physico-chemical characteristics *in situ* at each site concurrent with fish sampling. Water quality was measured using a calibrated Eutech PCD 650 multimeter (EUTECH Instruments Ltd, Singapore), and the variables measured include oxygen concentration and saturation, temperature, pH, electrical conductivity and total dissolved solids (TDS). We measured water clarity using a clarity tube (Kilroy and Biggs 2002).

During each fish survey, we collected subsurface water samples for laboratory analyses. Water samples were collected in clean polyethylene plastic bottles, ensuring no air bubbles were in the sample. Samples included a 2-l bottle (or 1-l × 2) for water quality analyses and a 500 ml bottle for microbial analyses. Once collected, the water samples were refrigerated at 4 °C (not frozen) until they were delivered to Umgeni Water’s Laboratory (Pietermaritzburg, South Africa) for analyses. The following variables were analysed: System variables (chemical oxygen demand (COD); electrical conductivity (mS m^−1^); alkalinity (CaCO_3_); turbidity (NTU)); salts (Chlorides (Cl); sulphates (SO_4_); calcium (Ca); sodium (Na)); nutrients (nitrates (NO_3_); nitrite (NO_2_); phosphorus (SRP and TP)); toxicants (ammonia (NH_3_)); microbial (Coliforms; *Escherichia coli*; heterotrophic plate count (HPC 37)); Chlorophyll a and fluorine (F).

### Habitat

We assessed habitat condition and availability using a visual scoring system that considers the relative availability of and scores the suitability of substrate, cover and flow biotope types (O’Brien et al. 2012). We measured velocity-depth using a transparent velocity head rod (Fonstad et al. 2005). We categorised the substrata available into different types, namely bedrock, boulders, cobbles, gravel, sand, mud and silt (Kleynhans 1999). Habitat parameters also included cover comprising undercut banks, root wads, marginal vegetation, overhanging vegetation, aquatic vegetation, substrata, depth/column (Kleynhans 1999) and woody debris.

### Multivariate statistical analyses

The use of multivariate statistical analysis techniques to evaluate biological communities in different ecosystems is common (Ter Braak 1994; O’Brien et al. 2009; Wepener et al. 2011). We used multivariate statistics to evaluate the response of fish assemblages to driving environmental variables (driver components), which included water quality and habitat. To analyse the data collected, we used a principal component analysis (PCA) approach (using CANOCO for Windows Version 4.53). The PCA is based on a linear response model relating species and environmental variables (van den Brink et al. 2003). The outcomes of the analysis (ordination) are represented as two-dimensional maps of the samples, where the placements of the samples indicate the (dis)similarities between samples (O’Brien et al. 2009). In this study, the samples in question were fish community samples based on the diversity and abundance of communities observed.

In addition to the PCA, we conducted various redundancy analyses (RDAs – a derivative of PCAs) to determine which species or environmental variables likely had the greatest influence on the structure or groupings reflected in the PCA. To do this, we overlaid the fish species and environmental variables (water quality and habitat) onto the original PCA. With the use of Canoco for Windows Version 4.53 (Ter Braak and Smilauer 2004), the RDA allows for the selection of the driving variables, which are then overlaid onto the PCA (O’Brien et al. 2009).

The RDA analyses use best-fit values (rather than the original data) estimated from a multiple linear regression between each variable in turn and a second matrix of complementary biological or environmental data (O’Brien et al. 2009). The outcome of the RDA was interpreted through two-dimensional bi-plots that indicated the similarities or dissimilarities between the samples (Shaw 2003; Van den Brink et al. 2003; O’Brien et al. 2009).

In the tri-plots containing the overlaid environmental and species data, each vector represented an environmental variable and points toward the steepest increase of values for the corresponding variable. The angles between vectors indicated the sign (+ or -) of the correlation between the variables; the approximated correlation is positive when the angle is less than 90° and negative when the angle is larger than 90° (O’Brien et al. 2009). The distance between the sampling sites in the diagram approximates the (dis) similarity of the variables as measured by their Euclidean distance (Shaw 2003). We transformed species data using a LogX^2^ transformation (because relative abundance data were available) (Van den Brink et al. 2003).

### Fish response assessment index (FRAI)

The FRAI is an assessment of the effect of environmental changes on fish communities to determine the well-being of said fish communities (Kleynhans 2007; Avenant 2010; Wepener et al. 2011). The index is specific to southern African freshwater ecosystems (Kleynhans 2007). The environmental variables used in FRAI and other similar indices usually include habitat integrity and water quality (Dickens and Graham 2002; Thirion 2007), together with a database of the intolerance and preference ratings for a variety of southern African freshwater species (Kleynhans 1999, 2003). The metrics that are assessed in FRAI are categories of these preferences and intolerances (Kleynhans and Louw 2007). The following metric categories assessed in FRAI (Kleynhans et al. 2005; Kleynhans 2007) include habitat availability (velocity-depth classes); flow modification (volume, timing and flow duration); migration; cover (undercut banks; overhanging vegetation; aquatic vegetation; water column (depth); substrata; root wads); physico-chemical metric (water quality); and introduced species.

Assessing the response of fish species to changing environmental conditions can either be done through direct measurement (surveys) or inferred from changing environmental conditions (habitat) (Kleynhans 2007). In this study, we conducted the assessment via direct measurement fish sampling. In FRAI, ecological responses are interpreted by linking changes in environmental conditions (drivers) to fish stress (Kleynhans 2007). The index is based on a combination of fish sample data and fish habitat data, in which the response of fish species to habitat changes is assessed based on knowledge of each fish species’ ecological requirements (preferences and intolerances) (Kleynhans 2007). The FRAI assessment develops a FRAI score out of 100 that relates to the ecological status or state of the sampled fish communities and is represented in ecological categories from A to F (Table 1; Kleynhans 2007). A combined ecological category, for example, a C/D and or a D/E, are given when the FRAI score is in the lower or upper end of an ecological category by two.

The FRAI results were in the form of both an automatic and an adjusted FRAI score. The automatic score is based solely on the differences in frequency of occurrence (FROC) between expected and observed fish species at each specific site (Kleynhans 2007). The expected FROC for fish species data were obtained from a historical database sourced from the Present Ecological State, Ecological Importance and Ecological Sensitivity (PESEIS) database (DWS 2014a), Ezemvelo KwaZulu-Natal Wildlife office, and the Global Biodiversity Information Facility (GBIF) as per Evans et al. (2022). However, the FROC assessment does not account for habitat (velocity-depth, cover, flow modification, and physico-chemical) and sampling effort, and hence, there is an adjusted score that can be manually altered to accommodate the variations in these factors. Manually adjusting the FRAI score allows the user to evaluate the state of environmental drivers according to each site’s habitat availability and sampling effort from collected habitat, water physico-chemical and other observed evidence that may impact the site. Adjustments were made using in-depth knowledge of the site, observations made during the surveys, and water quality parameters measured for each survey.

## Results

We collected a total of 14 fish species in this study (consisting of three assessments at each of the eight REMP sites and a total of 160 efforts). This resulted in a total abundance of 295 fish, of which *Labeobarbus natalensis* was the most common (*n* = 87), followed by *Pseudocrenilabrus philander* (*n* = 46) and *Tilapia sparrmanii* (*n* = 43). These three were the most common fish and were collected a75%, 50% and 63% of sites, respectively. Uncommon species included *Amphilius natalensis* (*n* = 6), *Awaous aeneofuscus* (*n* = 5), *Clarias gariepinus* (*n* = 2), *Enteromius anoplus* (*n* = 1), *Enteromius viviparus* (*n* = 1) and the invasive species *Oncorhynchus mykiss* (*n* = 1) (Table 2). The other invasive species collected in this study was *Micropterus nigricans* (*n* = 27). Unexpected indigenous species (*E. pallidus* and *E. viviparus*) were also collected at U2MGEN-LIONS and U2KARK-USMGN (Table 2).

### Multivariate statistical analyses

#### Fish communities based on site

**Figure 2:**
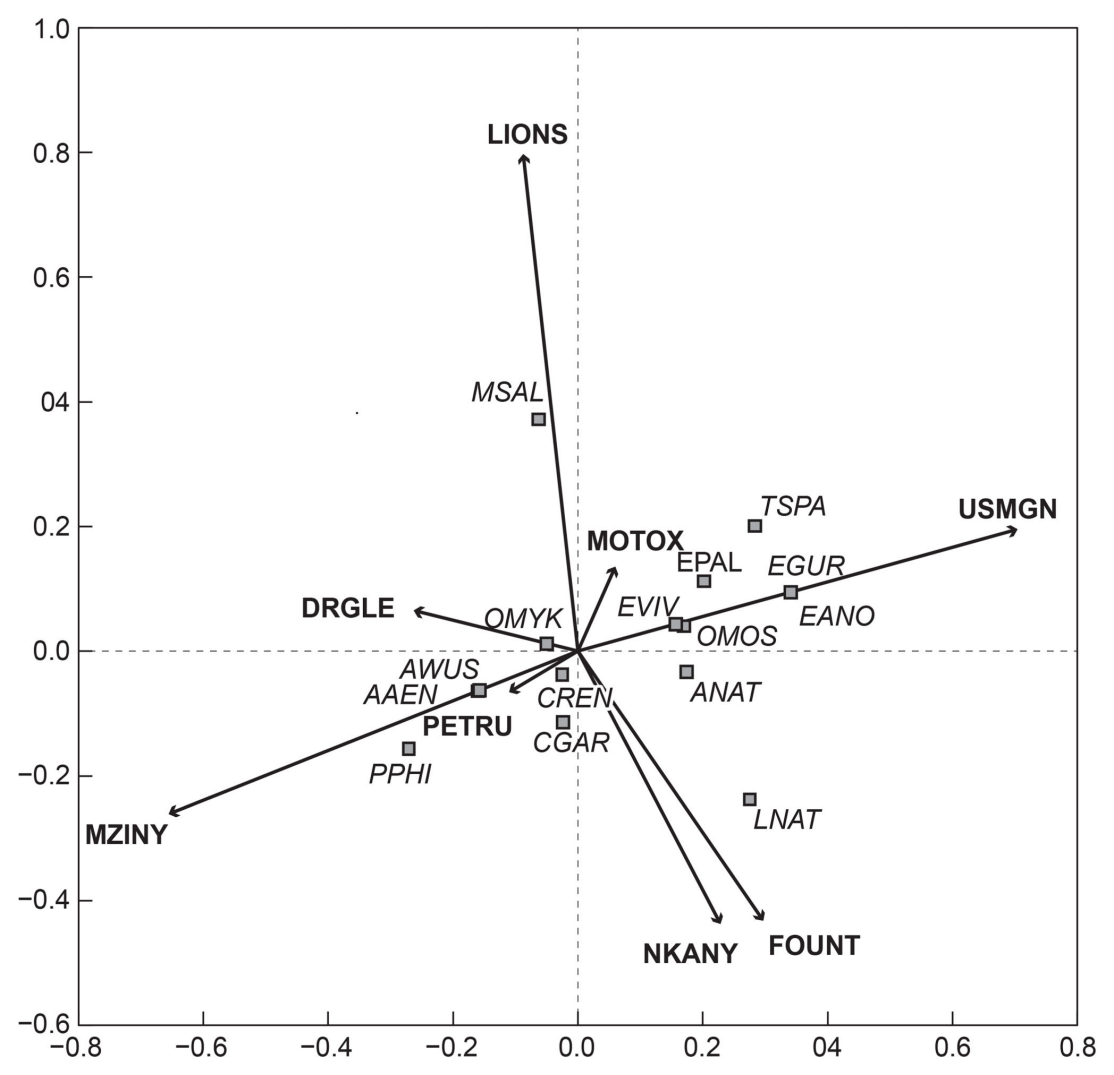
Redundancy analysis tri-plot of fish species and sites showing dissimilarity among sites (arrows) in the uMngeni River in the present study. The fish species (squares) were overlaid onto the RDA to show potential driving variables. (Abbreviations as per Table 2)

Redundancy analyses (RDA) of intersite community comparisons related to environmental variables, including water quality, quantity (flow) and habitat (Tables 3 and 4). Fish community structures at the various sites sampled in this study were significantly unique (*p* = 0.0010, Figure 2). A total of 65.5% of the total variation of data was presented in this ordination, with 39% of the variation on the first axis and 26.5% on the second axis (Figure 2). The only sites whose community structures were similar to one another were U2MGEN-PETRU and U2MGEN-MZINY (*p* = 0.062), as well as U2DUZI-NKANY and U2MGEN-FOUNT (*p* = 0.903, Figure 2). Site U2KARK-USMGN had the greatest species diversity, followed by U2MGEN-MZINY, which had a completely different fish assemblage. Invasive species *M. nigricans* (MSAL), and *O. mykiss* (OMYK) were positively associated with U2MGEN-LIONS and U2MGNI-DRGLE, respectively. *Labeobarbus natalensis* (LNAT) was associated with three (out of eight) sites in this study, namely, U2KARK-USMGN, U2DUZI-NKANY and U2MGEN-FOUNT. *Tilapia sparrmanii* (TSPA) was also associated with three sites: U2MGEN-LIONS, U2KARK-USMGN and U2DUZI-MOTOX.

#### Fish communities based on seasonal river flow (high flow vs. low flow)

**Figure 3:**
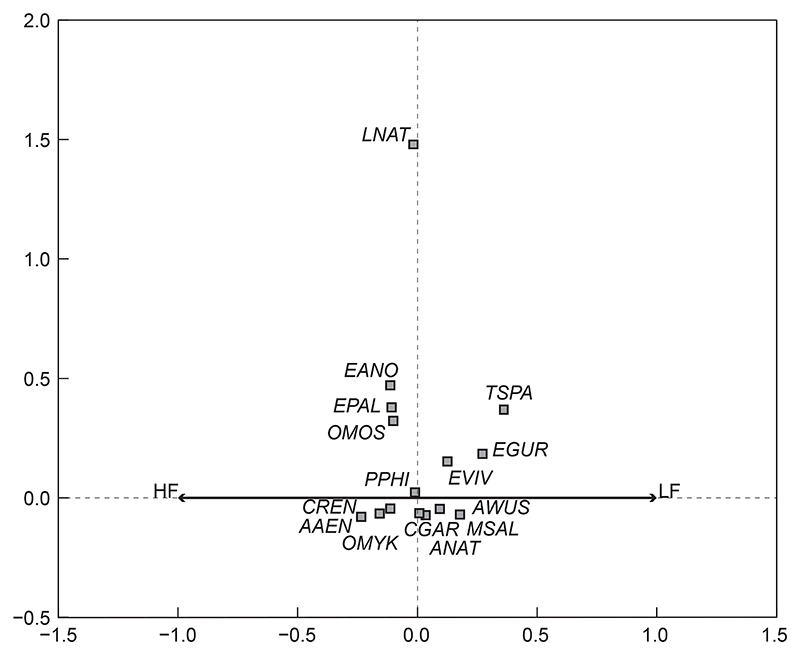
Redundancy analysis tri-plot of fish species, sites and flow showing dissimilarity between high and low flows (arrows) in the uMngeni River in the present study. The fish species (squares) and sites (triangles) were overlaid onto the RDA to show potential driving variables. (Abbreviations as per Table 2)

The results comparing fish community structures in high and low flow seasons indicated that fish community structures during the two flow seasons were significantly different in the uMngeni River in the present study (*p* = 0.0220, Figure 3), and thus, the flow had a significant influence on fish assemblages in this study. A 100% of the variation of data was presented in this ordination, all of which was on the first axis (Figure 3). Fish species that were associated with low flow seasons include *E. viviparus* (EVIV), *T. sparrmanii* (TSPA), *E. gurneyi* (EGUR) and *M. nigricans* (MSAL), while *E. anoplus* (EANO), *E. pallidus* (EPAL), *O. mossambicus* (OMOS), *C. rendalli* (CREN), *A. aeneofuscus* (AAEN) and *O. mykiss* (OMYK) were associated with high flow seasons. Species that were not necessarily related to either season included *L. natalensis* (LNAT), *P. philander* (PPHI), *C. gariepinus* (CGAR) and *A. natalensis* (ANAT), though the high flow season had slightly greater species diversity (Figure 3).

#### Fish communities based on velocity and depth

**Figure 4:**
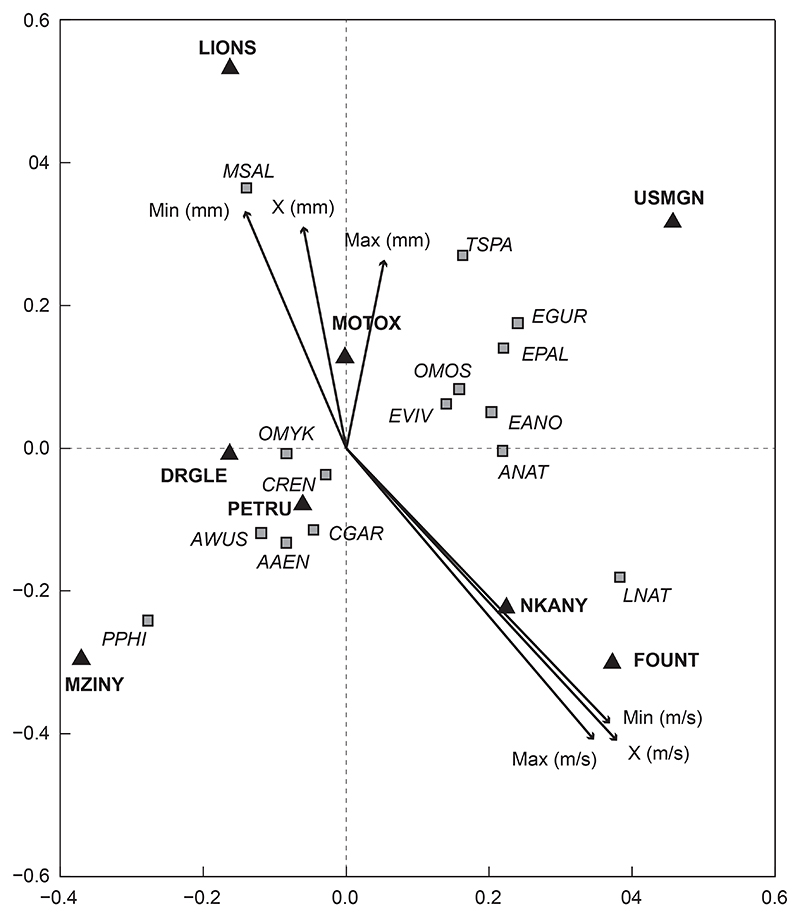
Redundancy analysis tri-plot of fish species, sites and velocity-depth showing dissimilarity between velocity and depth variables (arrows) in the uMngeni River in the present study. The fish species (squares) and sites (triangles) were overlaid onto the RDA to show potential driving variables. Note: Min (mm) is the minimum depth; Max (mm) is the maximum depth; X (mm) is the average depth; Min (m s^−1^) is the minimum velocity; Max (m s^−1^) is the maximum velocity and X (m s^−1^) is the average velocity. Other abbreviations as per Table 2

Velocity-depth was found to have a significant influence on fish community structure in the uMngeni River in the present study (*p* = 0.0090; Figure 4). In this ordination, 76% of the variation within the data was presented, with 48% of the variation on the first axis and 28% on the second (Figure 4). Mean velocity (X (m s^−1^)) accounted for the greatest variation (*F* = 4.15, *p* = 0.001). Maximum depth (max (mm); *F* = 2.19, *p* = 0.047) and minimum depth (min (mm); *F* = 3.60, *p* = 0.004) were also significant drivers of fish community structures in this study. Although some of the individual variables, namely minimum and maximum velocity and average depth, did not significantly influence fish community structure, there was a clear distinction between fish species that were more associated with depth variables and those that were more associated with velocity variables.

In this study, water depth was positively associated with species *M. nigricans* (MSAL), *T. sparrmanii* (TSPA), *O. mykiss* (OMYK), *E. gurneyi* (EGUR), *E. pallidus* (EPAL), *E. viviparus* (EVIV), *E. anoplus* (EANO), *A. natalensis* (ANAT) and *O. mossambicus* (OMOS) and sites U2MGEN-LIONS, U2KARK-USMGN and U2DUZI-MOTOX. Velocity was positively associated with species *A. natalensis* (ANAT), *E. anoplus* (EANO), *C. gariepinus* (CGAR), *A. aeneofuscus* (AAEN), and *C. rendalli* (CREN) and especially had a relatively strong positive correlation with *L. natalensis* (LNAT) and sites U2DUZI-NKANY and U2MGEN-FOUNT.

#### Fish communities based on substrate type

**Figure 5:**
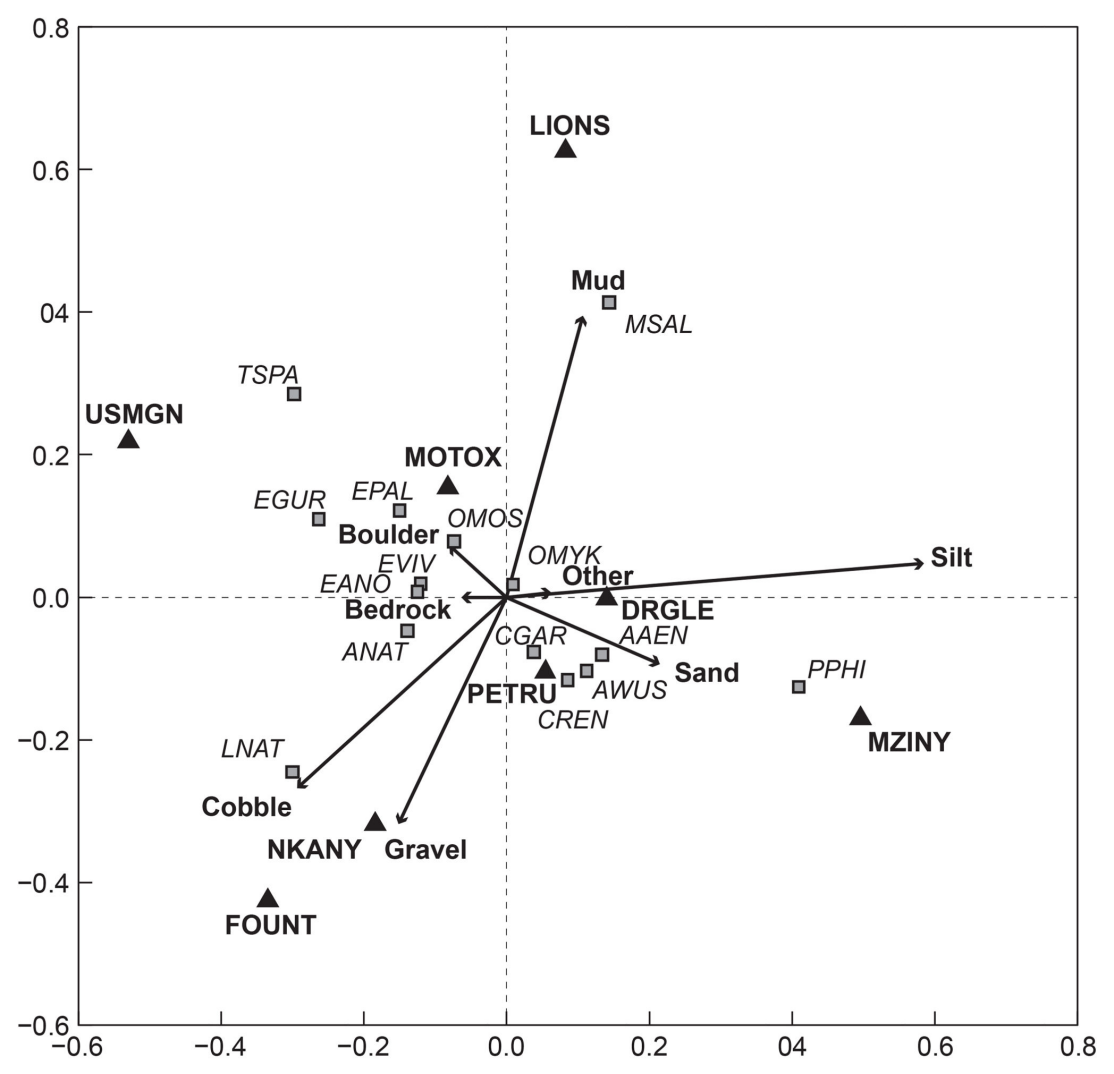
Redundancy analysis tri-plot of fish species, sites and substrate showing dissimilarity between substrate types (arrows) in the uMngeni River in the present study. The fish species (squares) and sites (triangles) were overlaid onto the RDA to show potential driving variables (abbreviations as per Table 2)

Statistical comparisons between fish communities and substrata found substrate to be a significant driver of fish community structures in the uMngeni River in the present study (*p* = 0.0380, Figure 5). In this ordination, 70.4% of the variation within the data was presented, with 49.5% of the variation on the first axis and 20.9% on the second axis (Figure 5). Of all the substrate types, silt accounted for the greatest variation in fish community structure (*F* = 5.16, *p* = 0.003), followed by mud (*F* = 2.76, *p* = 0.017), and cobbles (*F* = 2.15, *p* = 0.046).

*Awaous aeneofuscus* (AAEN), *P. philander* (PPHI) and sites U2MGNI-DRGLE and U2MGEN-MZINY were positively associated with silt. In contrast, other associations with silt included *C. gariepinus* (CGAR), *C. rendalli* (CREN), *M. nigricans* (MSAL) and sites U2MGEN-PETRU and U2MGEN-LIONS. Site U2MGEN-LIONS, *M. nigricans* (MSAL), and *O. mykiss* (OMYK) were positively associated with mud. Also associated with mud substrate was site U2DUZI-MOTOX and species *O. mossambicus* (OMOS), *E. pallidus* (EPAL), and *T. sparrmanii* (TSPA). Cobble substrate was positively associated with *L. natalensis* (LNAT), *A. natalensis* (ANAT) and sites U2MGEN-FOUNT and U2DUZI-NKANY. Also positively associated with cobble (to a lesser degree) were *E. viviparus* (EVIV), *E. anoplus* (EANO), *E. gurneyi* (EGUR), *E. pallidus* (EPAL), *T. sparrmanii* (TSPA) and site U2KARK-USMGN.

In addition to cobble substrate, *L. natalensis* (LNAT) was also positively associated with gravel, as were sites U2MGEN-FOUNT and U2DUZI-NKANY. *Pseudocrenilabrus philander* (PPHI), *A. aeneofuscus* (AAEN), *C. rendalli* (CREN) and sites U2MGEN-MZINY, U2MGEN-PETRU and U2MGNI-DRGLE were associated with sand (Figure 5). Boulder and bedrock substrate types both shared positive associations with *O. mossambicus* (OMOS), *E. pallidus* (EPAL), *T. sparrmanii* (TSPA), *E. gurneyi* (EGUR), *E. viviparus* (EVIV), *E. anoplus* (EANO), *A. natalensis* (ANAT) and sites U2DUZI-MOTOX and U2KARK-USMGN. Additionally, boulder substrate was positively associated with *M. nigricans* (MSAL) and U2MGEN-LIONS and bedrock was positively associated with *L. natalensis* (LNAT).

#### Fish communities based on cover feature type

**Figure 6:**
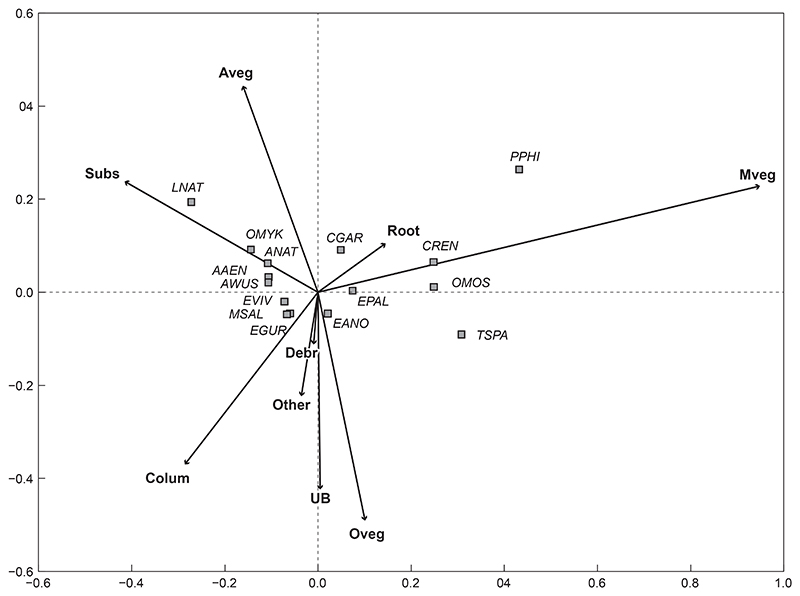
Redundancy analysis tri-plot of fish species and cover features showing dissimilarity between cover features (arrows) in the uMngeni River in the present study. The fish species (squares) were overlaid onto the RDA to show potential driving variables (abbreviations as per Table 2)

In the present study, cover features did not significantly influence fish community structure in the uMngeni River (*p* = 0.5330; Figure 6). This ordination showed 80.5% of the variation within the data, with 61% on the first axis and 19.5% on the second axis (Figure 6).

#### Fish communities based on water quality

**Figure 7:**
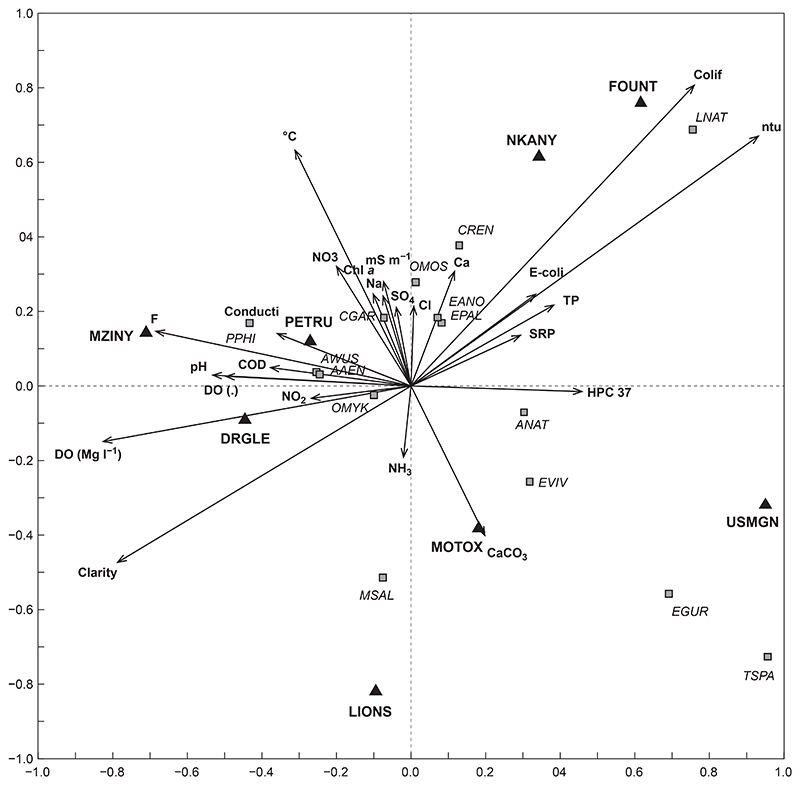
Redundancy analysis tri-plot of fish species, sites and substrate showing dissimilarity between water quality variables (arrows) in the uMngeni River in the present study. The fish species (squares) and sites (triangles) were overlaid onto the RDA to show potential driving variables (abbreviations as per Table 2)

In the present study, water quality was a significant positive driver of fish community structure in the uMngeni River (*p* = 0.0010, Figure 7). This ordination presented 57.2% of the variation within the data, with 32.7% of the variation on the first axis and 24.5% on the second axis (Figure 7). Numerous water quality variables that had a significant negative or positive influence on fish community structure included turbidity (NTU, *p* = 0.001), fluoride (F, *p* = 0.001), alkalinity (CaCO_3_, *p* = 0.035), sodium (Na, *p* = 0.016), heterotrophic plate count (HPC 37, *p* = 0.004), sulphate (SO_4_, *p* = 0.048), total phosphorus (TP, *p* = 0.031), conductivity (*p* = 0.031), nitrate (NO_3_, *p* = 0.001) and ammonia (NH_3_, *p* = 0.021).

The results showed that *L. natalensis* (LNAT) was positively associated with turbidity (ntu) and coliforms (Figure 7). *Oreochromis mossambicus* (OMOS) was positively associated with SO_4_, while *C. gariepinus* (CGAR) was positively associated with elevated Na (sodium), and *P. philander* (PPHI) was positively associated with conductivity (Figure 7). Sites U2DUZI-MOTOX and U2MGEN-MZINY were positively associated with CaCO_3_ and F, respectively (Figure 7).

### Fish Response Assessment Index (FRAI)

The assessment of the ecological integrity of fish assemblages using adjusted FRAI scores indicated a general downward trend from source/upper catchment to mouth/lower catchment, with FRAI Ecological Categories (ECs) ranging from moderately modified (C) to largely/severely modified (D/E) (Table 5).

#### Upper uMngeni River near Dargle (U2MGNI-DRGLE)

Indigenous species *Anguilla mossambica, A. natalensis, E. anoplus* and *L. natalensis* were expected in this region. However, none of the reference species for site U2MGNI-DRGLE were ever caught, and instead, a single *O. mykiss* was caught in the November 2017 (high flow) survey. The EC score at this site was C (moderately modified). The metric groups with the most weights in this site were the impact of introduced species, migration, and flow modification, indicating that they had the most influence on fish assemblages at this site (see Table 6).

#### Upstream of Midmar Dam (U2MGEN-PETRU)

According to the PESEIS reference species, the indigenous species *A. mossambica A. natalensis, E. anoplus* and *L. natalensis* are naturally occurring and expected in U2MGEN-PETRU (Skelton 2001; DWS 2014a). In the present study, however, only a single *L. natalensis* was caught in the May 2017 (high flow) survey. The adjusted EC score for this site was C (moderately modified). The metric groups with the most weights in this site were velocity-depth classes, flow-modification, and migration.

#### The Lions River tributary (U2MGEN-LIONS)

None of the expected reference fish species were caught in site U2MGEN-LIONS, but instead, invasive *M. nigricans* (more than any other site) and several indigenous fish species (*T. sparrmanii, E. pallidus* and *C. rendalli*) were caught over the course of this study. These were not on the expected species list for the site. The fish assemblage assessment (FRAI) outcome in the U2MGEN-LIONS site indicated that this site was largely modified (EC score was D). The top three metric groups identified to have the greatest impact on fish assemblage at this site were the impact of introduced species, flow modification, and migration.

#### The Karkloof River tributary (U2KARK-USMGN)

Based on the fish assemblage assessment (FRAI) outcome, the site U2KARK-USMGN was moderately modified (ecological category C). Seven out of ten reference species (DWS 2014a) were caught at this site (although the frequency of occurrence was lower than the reference), as well as two indigenous unexpected species, namely *E. pallidus* and *E. viviparus*, making this site the richest in both diversity and abundance over the course of this study (see Table 2). The fish assemblage assessment indicated that velocity-depth classes, flow modification, and physico-chemical characteristics (water quality) were the main drivers of change in the fish community at this site.

#### The uMngeni River mid-catchment (U2MGEN-FOUNT)

There were fifteen expected reference species at the U2MGEN-FOUNT site, and five of these species were caught over the duration of this study. Sampled fish species include *A. natalensis, L. natalensis, C. gariepinus, P. philander*, and *T. sparrmanii*, and all species had a low frequency of occurrence. No invasive or translocated species were caught at this site, and the FRAI assessment indicated that the site was moderately/largely modified (EC score was C/D). According to the FRAI assessment, velocity-depth classes, flow modification, and physico-chemical characteristics were the metric groups with the most weight at this site and, hence, the biggest drivers of change in fish community structure.

#### The Msunduzi River, Pietermaritzburg City (U2DUZI-MOTOX)

The fish assemblage assessment (FRAI) of U2DUZI-MOTOX indicated that this site was largely modified (EC score was D). Of the thirteen reference species that were expected at this site, only three (*L. natalensis, P. philander*, and *T. sparrmanii*) were found, and at lower frequencies of occurrence than the reference. Metric weights in the FRAI assessment indicated that velocity-depth classes, flow modification, and physico-chemical characteristics significantly influenced changes in the fish community structure of this site.

#### The Msunduzi River lower catchment (U2DUZI-NKANY)

There were thirteen expected reference fish species at this site, five of which were sampled over the course of this study. Sampled species include *L. natalensis, P. philander, C. rendalli, T. sparrmanii*, and the invasive species *M. nigricans*. Based on the outcome of the FRAI assessment of site U2DUZI-NKANY, this site was largely modified (EC score D), and metric weights indicated that flow modification, velocity-depth classes, and physico-chemical characteristics were the main drivers of change in fish community structure at this site.

#### The uMngeni River lower catchment (U2MGEN-MZINY)

Site U2MGEN-MZINY was the farthest down the uMngeni River and had the greatest number of expected reference fish species. There were 26 expected reference species at this site (DWS 2014a), and only seven of these were sampled (at low frequencies of occurrence) throughout the duration of the study, one of which is the invasive *M. nigricans*. Several reference fish species that were not sampled inhabit estuaries and freshwaters (Skelton 2001). The FRAI assessment of fish communities revealed that this site was largely modified (EC score D), and the May 2017 survey indicated it was seriously modified (EC score E). Metric weights at this site indicated that migration, flow modification, and velocity-depth classes had the most significant influence on changes in fish community structure at this site.

### Deteriorating trends in FRAI scores

Results of the application of the FRAI approach include a deteriorating trend in the state of the fish communities downstream along the Umgeni River. This trend decreased from a moderately modified fish community observed in the upper reaches of the catchment, which is associated with water quality and flow stressors because of land-use changes and alien invasive species that compete with and prey on indigenous species. The ecological integrity state of the fish deteriorated to a largely modified D state, and on occasion, in the Msunduzi River, the ecological integrity of the fish community has deteriorated to an unsustainable Severely Modified D/E and E state. This deterioration was representative of excessive changes to the water quality, flows, and river habitats, all associated with land-use changes. Indicator species identified through applying FRAI that were expected to be more common in the catchment included the five *Enteromius* spp. All of these fish species have a high preference for good water quality, which appears to be a major contributor to the deterioration of the FRAI scores.

## Discussion

The overall ecological integrity of the uMngeni was shown to be in a poor state. The low abundance and diversity of fish species showed this. When comparing other river assessments in South Africa faced with similar impacts and fish species, for example, the Crocodile (West) River (Levin et al. 2019), the Mvoti (O’Brien et al. 2009), the Harts-Vaal River (Malherbe et al. 2015), Elands River (O’Brien et al. 2014), and Msunduzi River (Ngcobo et al. 2025), the uMngeni River was in an overall generally poorer ecological state. In KwaZulu-Natal, the uMngeni River has been shown to be the most anthropogenically impacted river (Evans et al. 2022). The ecological assessment in this study showed that the highly stressed system negatively impacted the uMngeni River’s ecological integrity. Using multivariate statistics and FRAI to assess the impacts and drivers of fish communities provided insight into where the ecological integrity can be addressed within the catchment and what drives these fish communities to improve the system’s ecological integrity.

### Multivariate analyses

The eight sites that were sampled had unique fish community structures, and the high flow season had a greater species diversity, which was to be expected, as in other studies (Schlosser 1985). Of the eight sites, six had *L. natalensis* present, commonly used as an indicator of ecosystem well-being (Impson et al. 2008; Burnett et al. 2021), though in U2MGEN-MZINY only one *L. natalensis* was caught over the study period. *Labeobarbus natalensis* is not expected to occur in high altitudinal and cold waters (Crass 1964), possibly explaining why they were not caught at the sites U2MGEN-LIONS and U2MGNI-DRGLE in this study. Despite this, anglers fish for *L. natalensis* in the Lions River, with the Caversham Mill Waterfall being the upstream limit for the species (Crass 1964). Although *L. natalensis* was present in most of the sites sampled in this study, the low numbers in which it occurs are concerning, and given that it is a migratory species and tolerant of poor water quality (Karssing 2007), this is most likely a consequence of the uMngeni River being heavily regulated and impounded by large instream dams which makes it the most fragmented river in KwaZulu-Natal (Ramulifho 2015; O’Brien et al. 2019).

Velocity-depth parameters were found to influence fish community structure in this study. Decreased water velocity, for instance, would result in a shift in fish community structure as species such as *L. natalensis* and *A. natalensis* showed a preference for high velocity, which is to be expected (Skelton 2001; Karssing 2007; Evans et al. 2022). The community structure shifted to include species such as *M. nigricans*, which generally prefer slow-flowing water. The velocity-depth classes of habitat are greatly influenced by the flow dynamics of a river (Kleynhans 2007), which in turn is subject to regulation by dams (Poff et al. 1997; Dugan et al. 2010; Fouchy et al. 2018). Site U2MGEN-FOUNT included habitats with fast-flowing water and was associated with *L. natalensis*. In this study, deep waters were associated with *M. nigricans* and *O. mykiss*, both invasive species. Similar to the findings in this study, Gao et al. (2019) found that dam regulation in the Yangtze River (China) caused significant shifts in fish assemblages, and gradually increasing numbers of non-native fish, a trend also seen in Spring Grove Dam in KwaZulu-Natal (Burnett et al. 2023; 2024).

The presence of gravel, cobbles and boulders is ecologically important as numerous KwaZulu-Natal fishes rely on these substrata as breeding and feeding grounds (Skelton 2001) and also influenced fish assemblages in this study. For instance, *L. natalensis* preferred cobbles and gravel (which it uses to spawn) (Crass 1964; Karssing 2007; Burnett et al. 2021). Noticeably, sites U2MGEN-FOUNT and U2DUZI-NKANY were both associated with these substrates and so had the two highest *L. natalensis* abundances. Certain substrate types can also act as a form of cover for fish (such as *A. aeneofuscus*) to hide from predators (Skelton 2001; Smokorowski and Pratt 2007). *Awaous aeneofuscus* is known to bury itself in the sand for cover (Skelton 2001) and was seen in this study to be associated with this substrate. Gravel, cobbles and boulders all have the potential to act as cover for fish, but when these substrata are buried under fine silt or mud (as found in sites U2MGEN-LIONS and U2MGNI-DRGLE), they are no longer useful for cover (Kleynhans 2007). In a systematic review of studies that examined the effect of habitat alterations, Taylor et al. (2019) found substrate type (e.g., gravel, cobble) to significantly affect the abundance of substrate-spawning fish.

The increase of sediment in rivers is generally anthropogenically driven through agriculture, commercial forestry and urban development, such as dams, road construction and infrastructure (Waters 1995; Dugan et al. 2010; McIntyre et al. 2016). As sedimentation reduces fish biodiversity (Poff et al. 1997; Hall et al. 2011; Hohensinner et al. 2018), having these activities near a river harms ecosystem health. Importantly, anthropogenic sedimentation reduces dams’ life span and capacity and increases the risk of water insecurity (Obialor et al. 2019; Perera et al. 2022). Therefore, it negatively impacts not only fish community structures but also water security for the region. In the present study, water quality significantly influenced fish community structure, explained by 57.2 % of the variation. The indicator species *L. natalensis* showed a preference for waters of relatively high turbidity and was positively associated with microbial coliforms. None of the species preferred water clarity; this was expected as it can make fish more susceptible to predators (Skelton 2001; Figueiredo et al. 2016). Evans et al. (2022) found that invasive *Micropterus* spp. favour clearer water, and these are highly predatory piscivorous fishes (Burnett et al. 2023). Salts such as sulphates (SO_4_), sodium (Na) and chlorine (Cl) had an influence on fish community structure as species such as *O. mossambicus, C. gariepinus, C. rendalli, E. anoplus* and *E. pallidus* were associated with them. *Pseudocrenilabrus philander* also showed a preference for high conductivity, which is an indicator of ions in the water (DWAF 1996).

The sites U2MGEN-PETRU and U2MGEN-MZINY were also positively associated with elevated conductivity. One of the main sources of conductivity is sedimentation, which may result from run-off from agricultural activity (Walser and Bart 1999). Considering that the U2MGEN-PETRU site is surrounded by agricultural activity, its elevated electrical conductivity may be the reason. The effects of water quality changes on aquatic ecosystems have been studied extensively (Peters and Meybeck 2000; O’Brien et al. 2009; Peters 2009; DWS 2014b; Yavuzcan Yildiz 2017). Poor water quality often results in a decline in fish species, not only because of the intolerances of the fish but also because the organisms they feed on may decline (DWAF 1996; Bilotta and Brazier 2008). Unfavourable water quality conditions can also enhance the impact of invasive species, which are often more tolerant of deteriorated and polluted waters (Bunn and Arthington 2002; Dudgeon 2014; Gao et al. 2019).

### Fish response assessment index (FRAI)

Based on the outcome of the fish response assessment index (FRAI), the fish assemblage in the surveyed sections of the uMngeni River can be considered to be largely modified in the lower reaches and moderately modified in the upper reaches of the river. This is common in KwaZulu-Natal and many reaches of South Africa where resources are being developed (O’Brien et al. 2019). In addition, the upper catchment falls under the water source areas that are increasingly being protected to secure water (Le Maitre et al. 2018). In the eight sites surveyed for this study, a combined total of 26 indigenous reference riverine fish species were expected; of this, only 14 species (54%) were caught. This indicated poor detection of key indigenous fishes primarily associated with connectivity to the estuarine environment, for example, *A. aenofuscus*, which was expected upstream of iNanda Dam but did not occur in the study. Similarly, many expected riverine fish species were not detected downstream of the iNanda Dam. Alien invasive species (bass and trout) negatively impacted the presence of indigenous fish, and detecting lower abundances of expected species negatively impacted the FRAI score.

The site U2MGNI-DRGLE was shown to be moderately modified, as none of the four expected reference fish species were caught here. The invasive *O. mykiss* likely has the greatest influence on fish community structures at this site. *Oncorhynchus mykiss* inhabits cool (< 21 °C), clear and well-aerated waters and breeds in cold (< 15 °C) water flowing in winter (Skelton 2001), thus making U2MGNI-DRGLE a relatively suitable habitat for *O. mykiss*. This, together with the predatory nature of this species (Skelton 2001), is the likely reason it can completely dominate this site. The relatively good water quality (shown by the multivariate analysis to significantly influence fish community structure in this study) and minimal human modification in this site were the reasons for the fairly good adjusted FRAI score. Migration and flow modification were additional impacts that influenced the fish community structure at this site. Given the high dependence that the lifecycles of *A. mossambica, E. anoplus* and *L. natalensis* have on migration (Wallace et al. 1984; Skelton 2001; Impson et al. 2008). Natural and anthropogenic structures hinder the movement of *L. natalensis* into this reach of the uMngeni River (Crass 1964; Karssing et al. 2012), and are compounded by the presence of *O. mykiss* (Burnett et al. 2023). The presence of weirs as well as Midmar Dam downstream are the main contributors to the absence of *A. mossambica* (Hanzen et al. 2022). No collection of *A. natalensis* in the present study at this site is concerning and suggests a severe impact from *O. mykiss*. In addition, the flow alterations caused by debris from felled trees in some parts of this site may also play a role in the absence of *A. natalensis*, which is particularly intolerant of habitats with no flow (Crass 1964; Burnett et al. 2021). At the time of the study, the clearing of the invasive trees (*A. mearnsii*) caused a great disturbance to the river, with the intention of restoring riverine habitats in the future. Namugize et al. (2018) showed that land-use changes in the upper uMngeni catchment reduced natural vegetation by 17%, which has also influenced the river’s ecological state, including the state of fish (Jewitt et al. 2015).

Similarly, site U2MGEN-PETRU was found to be in a moderately modified state primarily because of modified flow conditions and barriers that hinder fish migration (i.e. the weir that is present at this site), both of which are detrimental to fish biodiversity (Dudgeon et al. 2006; Grill et al. 2019). The presence of *L. natalensis* at this site indicates that river connectivity from Midmar Dam is sufficient for this species. However, the lack of *A. mossambicus*, and *E. anoplus*, suggest migration is still hindered. *Amphilius natalensis* is a rheophilic species relatively intolerant of non-flowing waters (Marriott 1997; Skelton 2001). The presence of a weir at this site has modified natural flow patterns and impedes the fishes’ ability to migrate freely, thus resulting in a decline of fish species that should otherwise occur in high frequencies in this site.

Site U2MGEN-LIONS was shown to be largely modified, and according to the FRAI assessment, fish species with a high preference for flowing water and an affinity for migration decreased in frequency of occurrence. It can, therefore, be suspected that the decrease in fish community integrity at this site is primarily because of competition and predation by invasive fish, modified flow conditions and migration barriers. Additionally, invasive species (such as those found at this site) are more tolerant of unfavourable conditions such as increased temperatures and flow modifications (Bunn and Arthington 2002; Dudgeon 2014; Burnett et al. 2023). *Micropterus nigricans*, in particular, is tolerant of a wide temperature range (below 10 °C to 32 °C) and a wide range of feeding habits (Skelton 2001; Ellender and Weyl 2014), and so the increased presence of this invasive species is a further indication of this site’s deteriorated state. The preceding drought conditions may have played a role in the lack of reference species (Evans et al. 2022), especially semi-rheophilic and rheophilic species (such as *A. natalensis* and *L. natalensis*) and those whose life cycle relies on the ability to migrate (such as *A. mossambica, E. anoplus, L. natalensis, E. viviparus* and *C. gariepinus*) (Skelton 2001; DWS 2014a; Hanzen et al. 2019; Burnett et al. 2021). The unexpected species, particularly *T. sparrmanii* and *C. rendalli*, may be favoured by reduced flows. However, the absence of *C. gariepinus* in the present study is concerning.

Site U2KARK-USMGN was shown to be moderately modified, with velocity-depth classes, flow modification, physico-chemical, related to excessive nutrients, and sedimentations being the main drivers of change in fish community structure. These findings were further corroborated by the multivariate analysis performed in this study, which showed velocity-depth classes and physicochemical characteristics significantly influence fish community variation among the sites surveyed. The site had the greatest fish diversity despite a weir and several bridge crossings upstream, altering flows and velocity-depth classes from its natural state (Ramulifho 2015; O’Brien et al. 2019). Mud drove changes in fish communities for this site (according to the multivariate analysis). The elevated microbial levels (*E. coli*, coliforms and HPC 37) found in November could be attributed to the presence of a buffalo herd and other wild game that had recently frequented the site and are often found in the area, being in Karkloof Safari Spa (DWAF 1996; Zhu et al. 2019).

In the middle uMngeni catchment, site U2MGEN-FOUNT was moderately to largely modified according to the FRAI assessment. Species with a high preference for clear, flowing water decreased in frequency of occurrence compared with the reference. The metric groups with the most weight at this site were the velocity-depth classes, flow modification, and physicochemical characteristics. All these metrics are related to flow modification (Kleynhans 2007; Mantel et al. 2010; Jewitt et al. 2015) and can be attributed to the unnatural flow regime downstream of Albert Falls Dam (WRC 2002). Flow alterations can negatively affect habitats, sediment deposition, migration and life history/physiological cues such as fish recruitment and growth (Poff et al. 1997; Bunn and Arthington 2002; Hall et al. 2011). The water quality at this site was relatively good, with just slight elevations in microbial activity, namely *E. coli*, coliforms and HPC 37. This was congruent with the multivariate analysis that showed velocity-depth classes and physico-chemical characteristics were drivers of change in fish community structure in this study. Sand as a substrate was missing at this site, possibly impacting the presence of *A. aeneofuscus*, which uses the sand for cover (Skelton 2001; DWS 2014a). In addition, *A. aeneofuscus* species also requires connectivity to an estuary that may have limited its abundance with two major dams (Nagel Dam and iNanda Dam) that impacted the presence of catadromous migratory African freshwater eels. The low presence of vegetated pools at this site possibly impacted the presence of *E. viviparus, C. rendalii*, and *T. sparrmanii*, which prefer this habitat type (Skelton 2001).

Site U2DUZI-MOTOX, on the Msunduzi River, was shown to be largely modified and most influenced by changes in velocity-depth classes, flow modification, and physico-chemical characteristics. This site occurs in the most urbanised location in this study. The urbanised landcover and associated impacts, including weirs on the Msunduzi River, also affect the river’s flow and fish abundances and their migrations (Ngcobo et al. 2025). The consequences of urbanisation are most obvious at this site, and Levin et al. (2019) showed similar results by directly linking increased urbanisation to poor FRAI EC scores (i.e. ecological degradation). The Msunduzi River passes through the urban area of Pietermaritzburg, which further contributes to the pollution of the river (WRC 2002; Matongo et al. 2015), and site U2DUZI-MOTOX. This site was directly downstream of the Pietermaritzburg landfill site, adjacent to the river, influencing the natural substrate types available. Substrates were buried in solid anthropogenic waste, such as blankets, clothing, bags, etc., which heavily impacted the availability of substrates tested. This impacts *L. natalensis*, which requires gravel with no silt to breed (Crass 1964), and this was lacking at site U2DUZI-MOTOX. Extensive urbanisation, such as along the Msunduzi River, has been shown to alter flow and sedimentation, thus altering the habitat (Poff et al. 1997; Hall et al. 2011; Hohensinner et al. 2018). Other species, such as *A. natalensis*, live among cobbles, rocks and fast-flowing water (Skelton 2001), and *E. gurneyii* inhabits pools in clear, rocky streams (Skelton 2001) and is highly sensitive to water pollution. Both these species are now listed by the International Union for Conservation of Nature (IUCN) as vulnerable. The water quality at site U2DUZI-MOTOX was generally poor. The elevated chlorine concentrations may result from the Darvill Wastewater Treatment Works, less than 2 km upstream of this site. Chlorine has detrimental effects on fish and other river organisms (DWAF 1996). The high electrical conductivity at this site indicated high total dissolved salt concentration (i.e. ions in the water such as chloride, sulphate, nitrate, sodium, and calcium), indicative of an impacted site (DWAF 1996). Additionally, the ratio of nitrogen (NO_3_) to phosphorus (SRP) at this site was ~3:1, which is considered typical for environmental water impacted by raw sewerage (DWAF 1996), which was present at this site.

Similarly to site U2DUZI-MOTOX, the downstream Msunduzi River site U2DUZI-NKANY, was largely modified, and the FRAI metric weights indicated that flow modification, velocity-depth classes, and physico-chemical characteristics were the main drivers of change in fish community structure at this site. Species with a high preference for clear, flowing water decreased in frequency of occurrence. Multivariate analyses also indicated that velocity-depth classes and physico-chemical characteristics significantly influenced fish community variation among sites in this study. Once again, flow regulation was one of the main influences on the deteriorated state of this site. Alterations to the Msunduzi River’s natural flow are caused by the Henley Dam and associated weirs upstream of the site (Hall et al. 2011; Hohensinner et al. 2018). This site’s riparian zone (including marginal vegetation and banks) is also heavily deteriorated by anthropogenic activity, such as sand mining. Sand mining at this site has compromised the riverbank and riparian zone, which is detrimental to fish and other freshwater species (Padmalal et al. 2008; Kori and Mathada 2012; authors, pers. obs.). The riparian zone is important for maintaining freshwater biodiversity, and a compromised riparian zone leaves a lot of fish species without cover, especially juvenile fish (Skelton 2001; Pusey and Arthington 2003). Such species include *L. natalensis, C. rendalli, P. philander* and *E. pallidus*, most of which were absent or occurred in low frequencies at this site. The riparian zone is home to the invertebrates that fish feed on and also provides shade and cover for fish (Pusey and Arthington 2003). At this site, there is also the invasive aquatic plant *Eichhornia crassipes* (water hyacinth) which is scattered across the site. The dense mats that *E. crassipes* form can alter water quality, which has a detrimental effect on other aquatic life (Cilliers 1991; Fouchy et al. 2018). At this site, however, *E. crassipes* appeared not to have altered water quality (most likely because it does not occur in dense mats), and instead, water quality alterations appear to be a result of sand mining and cattle (authors, pers. obs.).

Site U2MGEN-MZINY, the lowest site on the uMngeni River in this study, was shown to be largely/severely modified with migration, flow modification, and velocity-depth classes having the most significant influence on changes in fish community structure. For the ecological scores from the FRAI assessment, fish species that migrate and have a high preference for flowing water decreased in frequency of occurrence. Flow alterations at this site are caused by the iNanda Dam, which also acts as a barrier for migratory fish moving upstream (Dugan et al. 2010; McIntyre et al. 2016). Migratory fishes depend on a range of habitats along a river ecosystem, so connectivity between habitats is important to maintain healthy biodiversity (O’Brien et al. 2019). The presence of *A. aeneofuscus* shows some connectivity to the estuary, with these fish being closely affiliated with the estuarine environment to complete their life cycle (Skelton 2001). The water quality below the iNanda Dam was relatively good because of the attenuation of water in the dam before it was released. The multivariate analyses indicated that the site was associated with high electrical conductivity, which is directly correlated with total dissolved salts concentration (i.e. ions in the water such as chloride, sulphate, nitrate, sodium, and calcium) (DWAF 1996). The site is also associated with high silt and sand deposits. Alterations in nutrient and sediment dynamics at this site may be a result of the flow alterations caused by low base flow releases from the iNanda Dam (Poff et al. 1997; Hall et al. 2011; Hohensinner et al. 2018).

## Conclusions

The results of the present study showed that the ecological integrity of the uMngeni River tends to degrade from the upper to lower reaches in response to various anthropogenic activities. The ecological categories of sites in the uMngeni River were largely driven by flow modifications and major fish migration barriers, for example, Midmar, Albert Falls, Nagel, and iNanda dams and weirs in the uMngeni that significantly impact the movement of diadromous species, for example, the African freshwater eels (Hanzen et al. 2022) and *A. aeneofuscus*. The presence and high abundance of invasive species also negatively impact the fish community structure at survey sites. The major dams and weirs regulate flows significantly, affecting the available habitat and the associated fish community structure at surveyed sites. This was compounded downstream in the uMngeni River (including the Msunduzi tributary) and shown in the ecological score. Importantly, the Msunduzi River sites consistently had an ecological score class of E, largely driven by the poor water quality, flow modification, and solid waste that have degraded the freshwater ecosystem here. Some expected remediation from stressors associated with Pietermaritzburg City at the U2DUZI-NKANY site was not present. Other associated stressors impacted this site, such as sand mining and residual water quality alterations from urban and rural activities. The application of the multi-metric index was successful, and the stressors driving fish communities were identified as being attributed to anthropogenic land-use activities. These outcomes conformed with the multivariate analyses that identified significant changes in communities.

Using multivariate analyses, the present study also showed that variations among the sites selected were significantly driven by changes in velocity-depth classes, substrate type and water quality (physico-chemical), all of which can be influenced by flow modifications. However, cover type was not a significant driver of fish community variations in this study. The outcomes include new evidence of altered fish communities associated with multiple stressors in the uMngeni catchment that need to be mitigated. We suggest that further consideration is given to managing instream barriers, water quality remediation, environmental flow requirements, and habitat restoration to improve the fish communities associated with this highly regulated and impacted river.

## Supplementary Material

**Supplementary material:** available online at https://doi.org/10.2989/16085914.2025.2564685

Supplementary figures

## Figures and Tables

**Table 1:** FRAI ecological category (EC) descriptions (Source: Kleynhans 2007)

Ecological categories	Name	Description	Acceptable/Unacceptable	Score (%)
A	Natural	Unmodified natural	Acceptable	90–100
B	Good	Mostly natural with few modifications	Acceptable	80–89
C	Fair	Moderately modified	Acceptable	60–79
D	Poor	Largely modified	Unacceptable	40–59
E	Seriously modified	Seriously modified	Unacceptable	20–39
F	Critically modified	Critically or extremely modified	Unacceptable	0–19

**Table 2:** Summary of fish species caught in the uMngeni River in the present study (May, August and November 2017) and their abbreviations. Translocated indigenous species are written in bold

Species name	Abbr.	U2MGNI-DRGLE	U2MGEN-PETRU	U2MGEN-LIONS	U2KARK-USMGN	U2MGEN-FOUNT	U2DUZI-MOTOX	U2DUZI-NKANY	U2MGEN-MZINY	Species abundance
Amphilius natalensis	ANAT	0	0	0	4	2	0	0	0	**6**
Awaous aeneofuscus	AAEN	0	0	0	0	0	0	0	5	**5**
*Clarias gariepinus*	CGAR	0	0	0	0	1	0	0	1	**2**
*Coptodon rendalli*	CREN	0	0	1	1	0	0	8	5	**15**
Enteromius anoplus	EANO	0	0	0	1	0	0	0	0	1
*Enteromius gurneyi*	EGUR	0	0	0	20	0	0	0	0	**20**
*Enteromius pallidus*	EPAL	0	0	2	26	0	0	0	0	**28**
*Enteromius viviparus*	EVIV	0	0	0	1	0	0	0	0	1
*Labeobarbus natalensis*	LNAT	0	5	0	11	21	5	44	1	**87**
*Micropterus nigricans* *	MSAL	0	0	23	0	0	0	2	2	**27**
*Oncorhynchus mykiss* *	OMYK	1	0	0	0	0	0	0	0	1
*Oreochromis mossambicus*	OMOS	0	0	0	10	0	0	0	3	**13**
Pseudocrenilabrus philander	PPHI	0	0	0	0	1	2	4	39	**46**
Tilapia sparrmanii	TSPA	0	0	10	13	3	13	4	0	**43**
**Abundance**		**1**	**5**	**36**	**87**	**28**	**20**	**62**	**56**	**295**
**Diversity**		**1**	**1**	**4**	**9**	**5**	**3**	**5**	**7**	**14**

* Invasive species in the study area

**Table 3:** Water physico-chemical characteristics (water quality) of the uMngeni River sites sampled in the present study (May, August and November 2017)

Sample period (month) and site	Temperature (°C)	pH	DO (Mg/l)	DO (%)	Conductivity (mS/m)	Clarity (cm)	Turbidity (ntu)	Alkalinity (mg CaCO3/L)	Cl (mg Cl/L)	NO_2_ (mg N/L)	NO_3_ (mg N/L)	SO4 (mg SO4/L)	Ca (mg Ca/L)	Chlorophyll a (μg/L)	COD (mg O2/L)	Coliforms (MPN/100mL)	E. coli (MPN/100mL)	F (μg F/L)	HPC 37 (cfu/mL)	Na (mg Na/L)	NH_3_ (mg N/L)	SRP (ug P/L)	TP (ug P/L)
MAY-U2MGEN-PETRU	16.1	6.94	8.7	-	5.95	88	4.2	25	2.8	0.1	0.1	1	5.3	0.8	20	579	83	100	269	5	0.1	5	111
MAY-U2DUZI-NKANY	20.5	6.93	7.23	85	30.7	21	38	58	31	0.34	4.94	15	23	2.6	20	24196	763	100	1000	29	0.45	208	373
MAY-U2MGEN-MZINY	20.8	7.1	9.74	101	190	80	2	37	0.1	0.22	15.4	14	0.6	20	1300	39	29.4	155	32.3	0.1	5	23	1.7
MAY-U2MGEN-LIONS	12.2	6.46	8.08	-	8.436	53	17	181	6.7	0.1	0.46	1	5.9	0.7	20	4839	775	100	318	7.5	0.1	17.2	32.1
MAY-U2MGEN-DRGLE	9.3	6.05	8	-	3.945	44	12	11	3.8	0.1	0.12	1.1	2.7	0.2	20	4839	1034	100	1000	4	0.1	5.49	57.3
MAY-U2MGEN-FOUNT	15	6.74	7.96	91	10.93	29	20	32	8.9	0.1	0.2	5.3	7.4	2.1	25	4839	300	100	421	11	0.1	21.1	82.5
AUG-U2MGEN-PETRU	13.8	7.26	7.99	76	7.14	90	2.5	30	3	0.1	0.18	1.6	4.8	0.4	20	261	101	100	55	2.9	0.1	5	20
AUG-U2MGEN-DRGLE	9.8	7.62	8.26	75	4	90	0.5	21	2.6	0.1	0.35	1.2	3.9	0.3	20	157	9	100	46	2.3	0.1	5	24.1
AUG-U2MGEN-FOUNT	17	7.21	7.55	-	8.7	36	34	31	9.2	0.1	0.38	5.5	4.4	1.7	20	2420	219	100	1000	5.9	0.1	12.6	62.3
AUG-U2DUZI-MOTOX	16.5	7.3	7.5	-	40.2	60	3.2	116	49.6	0.1	1	37	22	4	20	2420	72	100	1000	45	8.09	354	472
AUG-U2KARK-USMGN	13.6	7.12	7.95	77	8	65	6.2	38	6.1	0.1	0.29	2.1	5.6	1.3	20	308	36	100	1000	4.1	0.1	5	35.6
AUG-U2MGEN-LIONS	12.5	7.43	7.82	77	9.1	65	4.9	45	7.6	0.1	0.23	1.7	8	0.3	20	411	7	100	1000	6.2	0.1	5	27.6
AUG-U2DUZI-NKANY	14.1	6.93	7.96	-	36.3	50	9.2	70	47	0.1	7.03	36	20	35	20	2420	138	100	1000	39	0.89	241	350
AUG-U2MGEN-MZINY	19.4	7.89	7.95	86	25.7	95	1.6	70	36	0.1	0.83	17	19	1.5	20	387	15	149	1000	40	0.1	8.02	33.8
NOV-U2MGEN-MZINY	22.7	8.10	7.8	90.6	33.4	75	2.1	74	42	0.1	0.49	22	14	1.5	20	1414	2	163	1000	36	0.1	5	15
NOV-U6DUZI-MOTOX	19.6	7.37	8.18	89.4	42.7	55	3.2	100	50	0.1	1.04	34	20	4.1	20	2250	69	100	1000	40	7.3	335	460
NOV-U2DUZI-NKANY			8.03	91.3	38.6	29	9.1	69	47	9.1	7.12	35	19	35	20	2420	147	100	1000	39	0.98	241	350
22.3																							
8.25																							
NOV-U2MGEN-DRGLE	15.1	7.33	8.67	86.2	5.01	88	0.9	19	2.7	0.1	0.33	2	3.8	1	20	179	12	100	292	3.1	0.1	5	15
NOV-U2MGEN-LIONS	19	7.17	8.15	87.8	13.2	54	7.5	48	8.3	0.1	0.42	2.1	9.1	0.1	20	613	7	100	228	7.8	0.1	7.21	24.1
NOV-U2KARK-USMGN	19.7	7.33	8.02	87.6	9.5	49	6.8	35	5.3	0.1	0.42	3.1	6.3	0.3	20	2420	117	100	1000	5.5	0.1	5	16.5
NOV-U2MGEN-PETRU	18.7	6.0	8.23	88.3	6.46	76	4.5	26	3	0.1	0.19	1.9	5	0.7	20	4352	1233	100	1000	4.2	0.1	5	20

**Table 4:** Overall (average) habitat features of the uMngeni River sites sampled in the present study (May, August and November 2017)

Sample period (month) and site	Depth				Velocity					Substrate									Cover				
	Average depth (mm)	Min depth (mm)	Max depth (mm)	Average velocity (m/s)	Min velocity (m/s)	Max velocity (m/s)	Silt (%)	Mud (%)	Sand (%)	Gravel (%)	Cobble (%)	Boulder (%)	Bedrock (%)	Other (%)	Undercut bank (%)	Roots (%)	Marginal veg (%)	Overhanginh veg (%)	Substrate (%)	Column (%)	Debris (%)	Aquatic veg (%)	Other (%)
MAY-U2MGEN-PETRU	464	405	505	-	0	-	0	0	0	2.5	5	45	48	0	0	0	0	0	70	27.5	2.5	0	0
MAY-U2DUZI-NKANY	275	136	410	0.5	0.3	0.6	0	0	6	28	57	9	0	0	0	0	14	1	52	33	0	0	0
MAY-U2MGEN-MZINY	416	329	494	0.3	0.2	0.4	14	7.5	19	0	2.5	30	24	3.8	0	0	26	0	58.8	12.5	0	0	2.5
MAY-U2MGEN-LIONS	359	293	460	0.2	0.2	0.3	0	2.5	0	7.5	39	50	0	1.3	0	0	28	11.3	46.3	17.5	0	0	0
MAY-U2MGEN-DRGLE	542	473	655	0.6	0.4	0.7	0	10	0	1.3	7.5	79	0	2.5	5	0	1.3	8.75	36.3	48.8	0	0	0
MAY-U2MGEN-FOUNT	351	246	453	0.4	0.3	0.6	0	0	0	5	5.7	86	3.6	0	0	0	5.7	0	66.4	27.9	0	0	0
AUG-U2MGEN-PETRU	464.3	356	556	0.2	0.1	0.2	0	0	1.4	5.7	2.9	37	53	0	0	0	0	0	64.3	34.3	1.43	0	0
AUG-U2MGEN-DRGLE	393	333	468	0.2	0.1	0.3	0	6.3	16	13	8.1	51	5	0	3.8	3.75	0	1.25	53.8	2.5	35	0	0
AUG-U2MGEN-FOUNT	407	332	483	0.3	0.2	0.3	0	17	0	10	3.3	30	40	0	0	0	37	0	21.7	28.3	13.3	0	0
AUG-U2DUZI-MOTOX	290	213	383	0.2	0.1	0.3	0	2.2	0.6	0.6	0	11	86	0	0	0	28	5.56	0	44.4	0	0	22
AUG-U2KARK-USMGN	433	357	512	0.1	0.1	0.1	0	27	0.5	4	0	69	0	0	3	0	16	0	46.5	29	6	0	0
AUG-U2MGEN-LIONS	524	421	614	0	0	0.1	14	19	0	0	2.7	52	13	0	0	0	12	0	45.9	41.8	0	0	0
AUG-U2DUZI-NKANY	302	219	376	0.4	0.2	0.5	1	2	6	28	47	16	0	0	0	0	0	0	59.5	24.5	0	16	0
AUG-U2MGEN-MZINY	408	327	493	0.2	0.1	0.3	6	22	14	1	9	37	11	0.7	0	0	27	0	35.7	35.7	0	1.3	0
NOV-U2MGEN-MZINY	312.0	236	358	0.23	0.07	0.33	6	2	16	4	0	46	13	13	0	0	38	0	38	16	0	0	8
NOV-U2DUZI-MOTOX	329.6	257.1	284.3	0.13	0.05	0.20	7.86	2.86	0	0	0	0	85	4.29	7.14	11.43	20	14.29	15.71	12.86	11.43	0	7.14
NOV-U2DUZI-NKANY	365.0	258	462	0.4	0.3	0.4	2	20	3	1	24	50	0	0	0	0	20	0	41	28	3	8	0
NOV-U2MGEN-DRGLE	312.3	263	351	0.3	0.2	0.4	12	11	9.6	0	11	43	14	0	8.3	0	0	5.83	40	15	25	0	5.8
NOV-U2MGEN-LIONS	270.4	187	340	0.3	0.3	0.4	13	0	0	0	7.5	18	61	0	6.7	0	28	11.7	30.8	20.8	1.67	0	0
NOV-U2KARK-USMGN	526.6	391	684	0	0	0.1	0	16	14	0	0	69	0	0	0	1.25	28	0	37.5	34.4	1.25	0	0
NOV-U2MGEN-PETRU	685.0	540	848	0.1	0	0.2	15	34	0	0	1.3	50	0	0	0	0	18	0	36.3	46.3	0	0	0

**Table 5:** Adjusted FRAI scores and ecological categories (EC) of uMngeni River REMP sites. A C/D and or a D/E are given when the FRAI score is in the lower or upper end of an EC by two

Site name	May 2017		Aug 2017		Nov 2017	
	FRAI score	EC	FRAI score	EC	FRAI score	EC
U2MGNI-DRGLE	74.3	C	75.6	C	69	C
U2MGEN-PETRU	62.3	C	69.5	C	69.7	C
U2MGEN-LIONS	48.3	D	46.9	D	48.7	D
U2KARK-USMGN	—	—	70.4	C	69.6	C
U2MGEN-FOUNT	61.1	C/D	53.4	D	—	—
U2DUZI-MOTOX	—	—	47.6	D	43.4	D
U2DUZI-NKANY	54.0	D	44.1	D	40.3	D/E
U2MGEN-MZINY	37.2	E	44.1	D	44.0	D

**Table 6:** Weights of metric groups used in the Fish Response Assessment Index (FRAI); these are developed scores that weighted the influence of the respective habitat type and condition into the FRAI-adjusted score in the present study

Metric group weights (%)	Velocity-depth	Cover	Flow modification	Physico-chemical	Migration	Impact of introduced
U2MGNI-DRGLE	75.0	73.4	76.6	68.8	81.3	100.0
U2MGEN-PETRU	100.0	71.9	94.7	86.0	94.7	64.9
U2MGEN-LIONS	81.7	68.3	98.3	55.0	90.0	100.0
U2KARK-USMGN	100.0	78.6	100.0	89.3	78.6	82.1
U2MGEN-FOUNT	100.0	78.6	100.0	92.9	82.1	75.0
U2DUZI-MOTOX	100.0	75.0	100.0	89.3	85.7	78.6
U2DUZI-NKANY	100.0	77.8	100.0	92.6	88.9	88.9
U2MGEN-MZINY	92.9	78.6	96.4	78.6	100.0	82.1
