# Supplementary material for: Assessment of the ecological integrity and fish community structures of the uMngeni River, KwaZulu-Natal, South Africa
Source: Afr J Aquat Sci. Author manuscript; Available in PMC 2025 Dec 25. (PMC7618539; doi:10.2989/16085914.2025.2564685)
Supplement: Supplementary figures [file EMS211452-supplement-Supplementary_figures.pdf]

SUPPLEMENTARY MATERIAL

African Journal of Aquatic Science, 2025

https://doi.org/10.2989/16085914.2025.2564685

Assessment of the ecological integrity and fish community structures of the uMngeni River, KwaZulu-Natal, South Africa

Pumla Dlamini[1], Colleen T Downs[1]*, Matthew Burnett[1] and Gordon O'Brien[1,3]

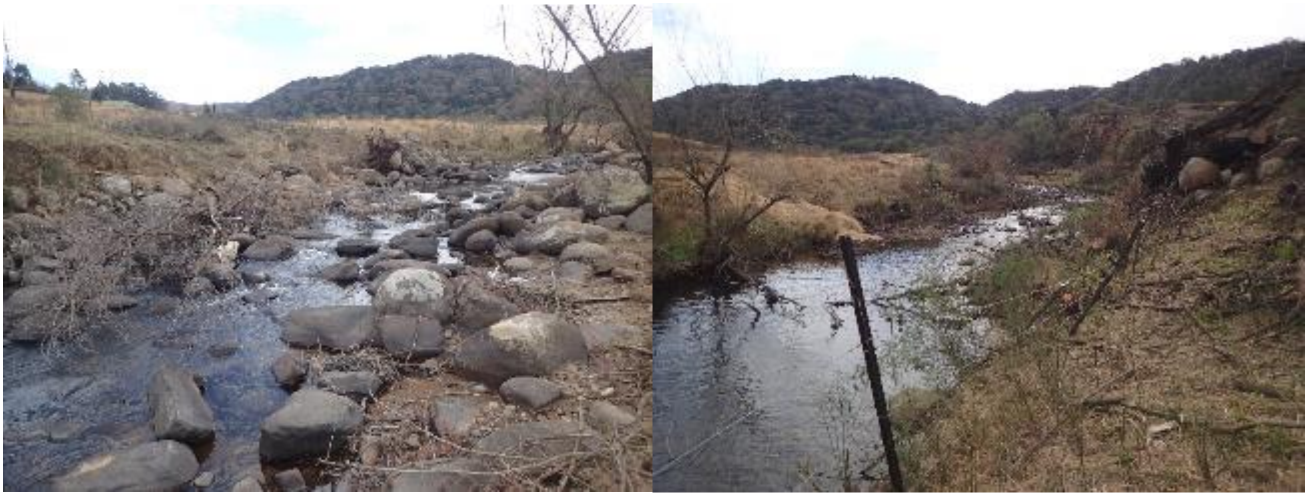

Supplementary Figure S1a: Photographs of site U2MGNI-DRGLE

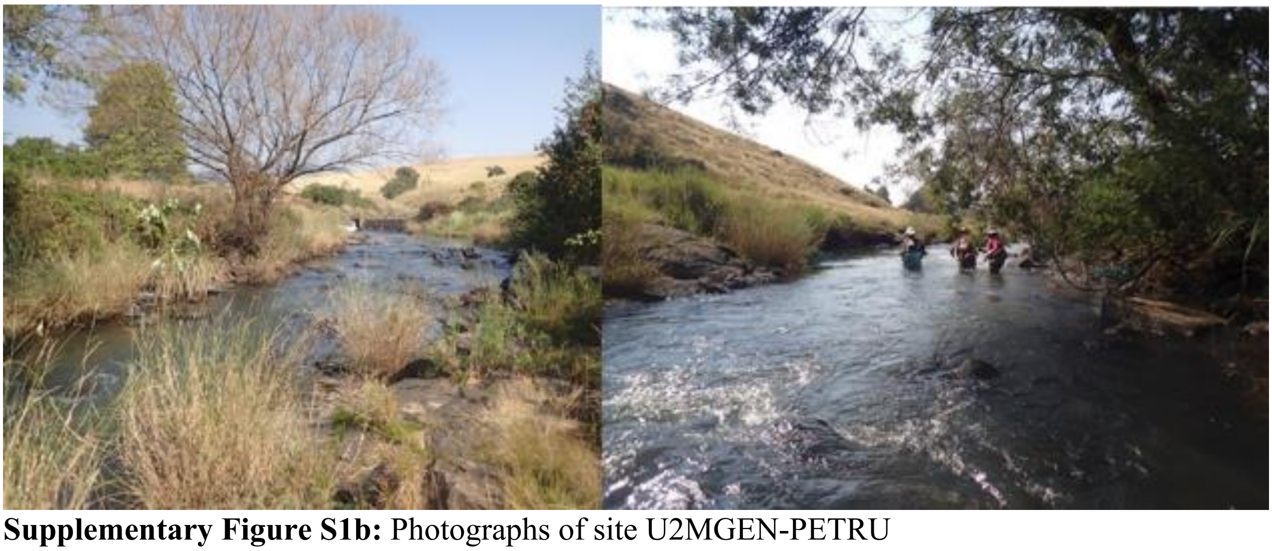

Supplementary Figure S1b: Photographs of site U2MGEN-PETRU

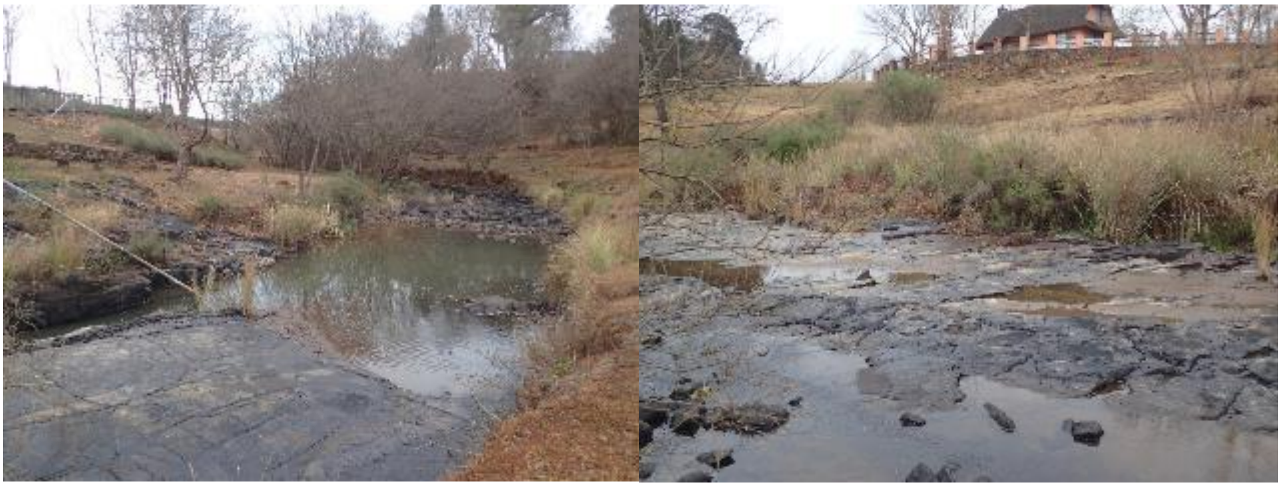

Supplementary Figure S1c: Photographs of site U2MGEN-LIONS

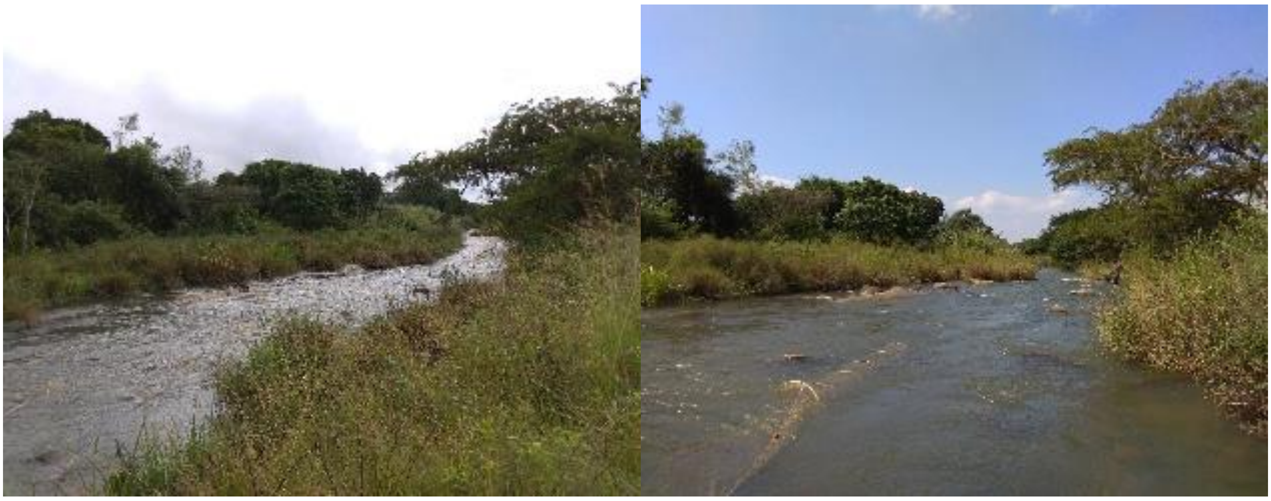

Supplementary Figure S1d: Photographs of site U2KARK-USMGN

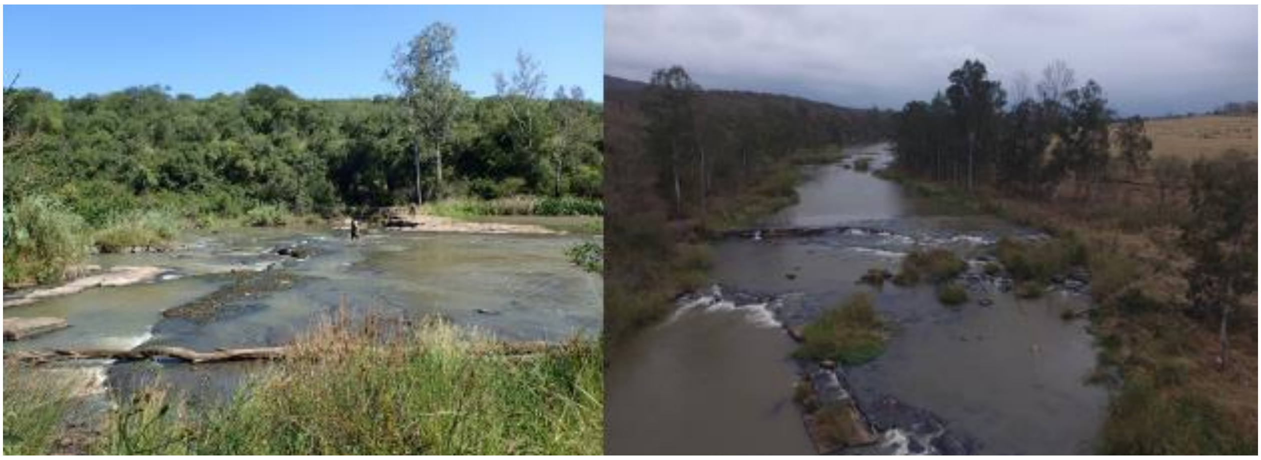

Supplementary Figure S1e: Photographs of site U2MGEN-FOUNT

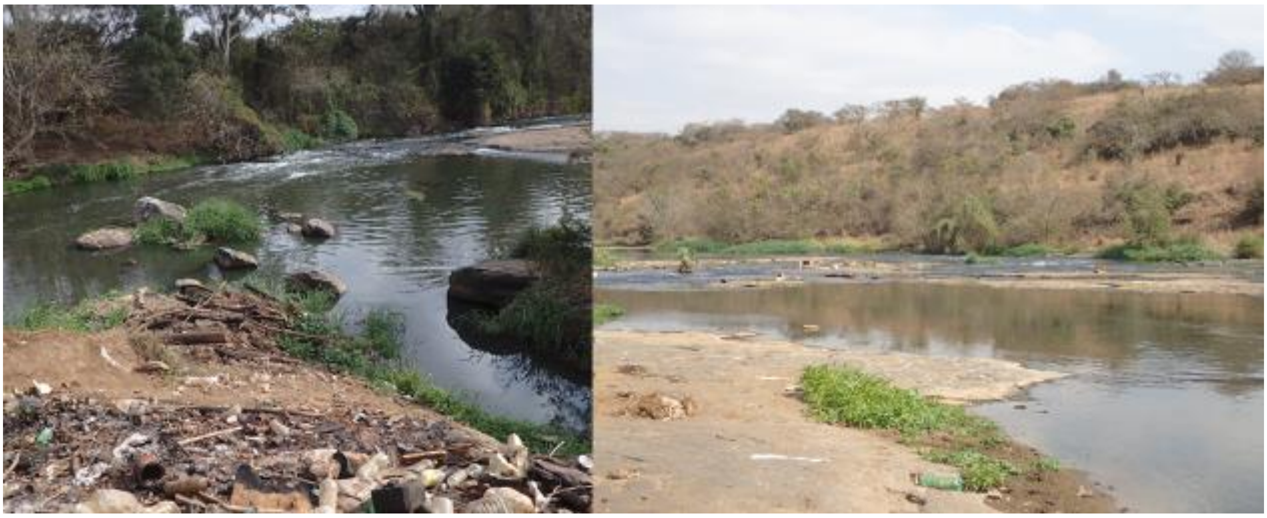

Supplementary Figure S1f: Photographs of site U2DUZI-MOTOX

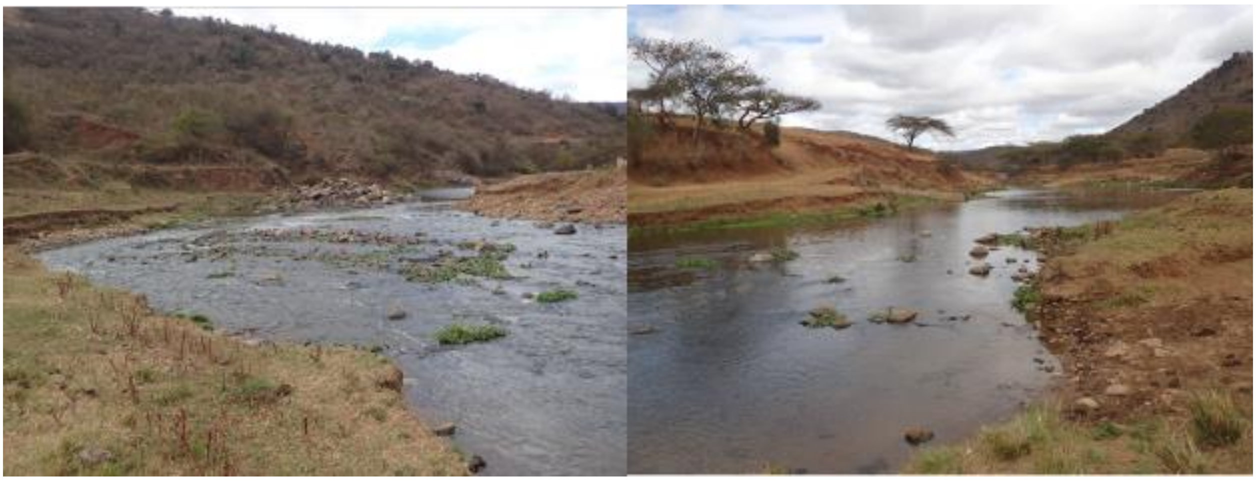

Supplementary Figure S1g: Photographs of site U2DUZI-NKANY

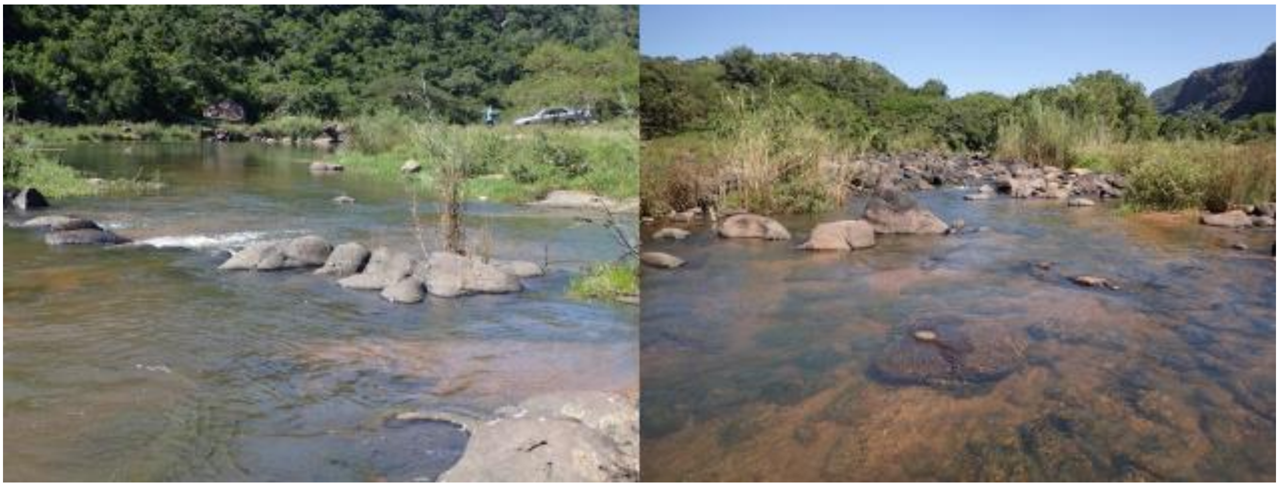

Supplementary Figure S1h: Photographs of site U2MGEN-MZINY

Supplementary Table S1: River Eco-status Monitoring Programme (REMP) sites sampled and their coordinates in the present study

REMP site name	River	Latitude	Longitude
U2MGNI-DRGLE	uMngeni	-29.488805	29.903036
U2MGEN-PETRU	uMngeni	-29.512469	30.094401
U2MGEN-LIONS	Lions	-29.414572	30.094375
U2KARK-USMGN	Karkloof	-29.443797	30.319403
U2MGEN-FOUNT	uMngeni	-29.491252	30.492632
U2DUZI-MOTOX	Msunduzi	-29.607	30.4508
U2DUZI-NKANY	Msunduzi	-29.611	30.5578
U2MGEN-MZINY	uMngeni	-29.720833	30.903937

Supplementary Table S2 {This table is not referenced in the main text}: FRAI ecological categories (Kleynhans 2007) and justification sheets for each survey conducted over the course of this study. Lists of expected species were taken from the Department of Water and Sanitation's PESEIS documents (DWS 2014a)

Ecological categories	Name	Description	Acceptable/ Unacceptable	Score (%)
A	Natural	Unmodified natural	Acceptable	90–100
B	Good	Mostly natural with few modifications	Acceptable	80–89
C	Fair	Moderately modified	Acceptable	60–79
D	Poor	Largely modified	Unacceptable	40–59
E	Seriously modified	Seriously modified	Unacceptable	20–39
F	Critically modified	Critically or extremely modified	Unacceptable	0–19

**Site Name**	U2MGNI-DRGLE	**Assessor**	P Dlamini
**River**	uMngeni	**Reviewed**	G O'Brien

ABR	SPECIES	REFERENCE FROC	OBSERVED FROC		
			May	Aug	Nov
AMOS	ANGUILLA MOSSAMBICA PETERS 1852	5.00	0	0	0
ANAT	AMPHILIUS NATALENSIS BOULENGER. 1917	5.00	0	0	0
BANO	BARBUS ANOPLUS WEBER. 1897	5.00	0	0	0
BNAT	BARBUS NATALENSIS CASTELNAU. 1861	3.00	0	0	0

Response of species with a preference/tolerance to		May	Aug	Nov
Velocity Depth Metric	Fast-Deep	-2	-2	-1
	Fast-Shallow	-2	-2	-2
	Slow-Deep	-1	-1	-1
	Slow-Shallow	0	0	0
Cover features	Overhanging veg	2	3	3
	Undercut banks	2	1	1
	Substrate	0	0	0
	Instream veg	3	1	3
	Water column	1	1	2

Response of species that are		May	Aug	Nov
Flow dependence	Intolerant to no flow	-2	-2	-2
	Moderately intolerant to no flow	-1	-1	-1
	Moderately tolerant to no flow	-1	-1	-1
	Tolerant to no flow	0	0	0

Response of species that are		May	Aug	Nov
Physico-chemical conditions	Intolerant to modified physico-chemical conditions	-1	-1	-1
	Moderately intolerant to modified physico-chemical conditions	0	0	0
	Moderately tolerant to modified physico-chemical conditions	-1	-1	-1
	Tolerant modified to physico-chemical conditions	0	0	0

Response of which require		May	Aug	Nov
Migration	Catchment scale movement	2	2	2
	Movement between reaches	2	2	2
	Movement within a reach	0	0	0

Extent of the following in the reach		May	Aug	Nov

		May	Aug	Nov
Changes in connectivity	Weirs and causeways	2	2	2
	Impoundments	2	2	2
	Physico-chemical barriers	1	1	1
	Flow modifications	1	1	1
Introduced/alien species		May	Aug	Nov
Introduced/alien species	Introduced/alien predacious species 1	-	-	OMYK
	Introduced/alien predacious species 2	-	-	-
	Introduced/alien predacious species 3	-	-	-
	Introduced/alien habitat modifying species 1	-	-	-
	Introduced/alien habitat modifying species 2	-	-	-
	The impact of introduced competing spp?	0	0	5
	FROC of introduced competing spp?	0	0	0.5
	The impactof introduced habitat modifying spp?	0	0	0
	FROC of habitat modifying spp?	0	0	0
**AUTOMATED FISH RESPONSE ASSESSMENT INDEX SCORE**				
FRAI (%)		16.6	15.3	9.5
EC: FRAI		F	F	F
**ADJUSTED FISH RESPONSE ASSESSMENT INDEX SCORE**				
FRAI (%)		74.3	75.6	69
EC: FRAI		C	C	C

**Site Name**	U2MGEN-PETRU	**Assessor**	P Dlamini
**River**	uMngeni	**Reviewed**	G O'Brien

ABR	SPECIES	REFERENCE FROC	OBSERVED FROC May	Aug	Nov
AMOS	ANGUILLA MOSSAMBICA PETERS 1852	5.00	0	0	0
ANAT	AMPHILIUS NATALENSIS BOULENGER. 1917	5.00	0	0	0
BANO	BARBUS ANOPLUS WEBER. 1897	5.00	0	0	0
BNAT	BARBUS NATALENSIS CASTELNAU. 1861	3.00	0	1	0

Response of species with a preference/tolerance to		May	Aug	Nov
Velocity Depth Metric	Fast-Deep	-2	-1	-1
	Fast-Shallow	-2	-2	-1
	Slow-Deep	0	0	0
	Slow-Shallow	0	0	0

Cover features	Overhanging veg	1	1	1
	Undercut banks	2	2	2
	Substrate	3	2	3
	Instream veg	2	2	2
	Water column	2	1	1
Response of species that are		May	Aug	Nov
Flow dependence	Intolerant to no flow	-2	-2	-2
	Moderately intolerant to no flow	-1	-1	-1
	Moderately tolerant to no flow	-1	-1	-1
	Tolerant to no flow	0	0	0
Response of species that are		May	Aug	Nov
Physico-chemical conditions	Intolerant to modified physico-chemical conditions	-3	-2	-2
	Moderately intolerant to modified physico-chemical conditions	0	0	0
	Moderately tolerant to modified physico-chemical conditions	-2	-2	-2
	Tolerant modified to physico-chemical conditions	0	0	0
Response of which require		May	Aug	Nov
Migration	Catchment scale movement	3	3	3
	Movement between reaches	3	3	3
	Movement within a reach	1	1	1
Extent of the following in the reach		May	Aug	Nov
Changes in connectivity	Weirs and causeways	4	4	4
	Impoundments	2	2	2
	Physico-chemical barriers	1	1	1
	Flow modifications	1	1	1
Introduced/alien species		May	Aug	Nov
Introduced/alien species	Introduced/alien predacious species 1	-	-	-
	Introduced/alien predacious species 2	-	-	-
	Introduced/alien predacious species 3	-	-	-
	Introduced/alien habitat modifying species 1	-	-	-
	Introduced/alien habitat modifying species 2	-	-	-
	The impact of introduced competing spp?	0	0	0
	FROC of introduced competing spp?	0	0	0
	The impact of introduced habitat modifying spp?	0	0	0
	FROC of habitat modifying spp?	0	0	0

AUTOMATED FISH RESPONSE ASSESSMENT INDEX SCORE			
FRAI (%)	12.6	12.7	7.5
EC: FRAI	F	F	F
**ADJUSTED FISH RESPONSE ASSESSMENT INDEX SCORE**			
FRAI (%)	62.3	69.5	69.7
EC: FRAI	C	C	C

**Site Name**	U6MGEN-LIONS	**Assessor**	P Dlamini
**River**	uMngeni	**Reviewed**	G O'Brien

ABR	SPECIES	REFERENCE FROC	OBSERVED FROC		
			May	Aug	Nov
AMOS	ANGUILLA MOSSAMBICA PETERS 1852	3.00	0	0	0
ANAT	AMPHILIUS NATALENSIS BOULENGER. 1917	3.00	0	0	0
BANO	BARBUS ANOPLUS WEBER. 1897	5.00	0	0	0
BGUR	BARBUS GURNEYI GÜNTHER. 1868	3.00	0	0	0
BNAT	BARBUS NATALENSIS CASTELNAU. 1861	3.00	0	0	0
BVIV	BARBUS VIVIPARUS WEBER. 1897	3.00	0	0	0
CGAR	CLARIAS GARIEPINUS (BURCHELL. 1822)	3.00	0	0	0

Response of species with a preference/tolerance to		May	Aug	Nov
Velocity Depth Metric	Fast-Deep	-1	-1	-1
	Fast-Shallow	-2	-1	-1
	Slow-Deep	-3	-3	-3
	Slow-Shallow	-3	-3	-3
Cover features	Overhanging veg	1	3	3
	Undercut banks	2	3	3
	Substrate	3	3	3
	Instream veg	3	3	3
	Water column	2	0	2
**Response of species that are**		**May**	**Aug**	**Nov**
Flow dependence	Intolerant to no flow	-4	-4	-4
	Moderately intolerant to no flow	-3	-3	-3
	Moderately tolerant to no flow	-2	-2	-2
	Tolerant to no flow	-1	0	0
**Response of species that are**		**May**	**Aug**	**Nov**

Physico-chemical conditions	Intolerant to modified physico-chemical conditions	-1	-1	-1
	Moderately intolerant to modified physico-chemical conditions	-1	0	0
	Moderately tolerant to modified physico-chemical conditions	0	0	0
	Tolerant modified to physico-chemical conditions	0	0	0
Response of which require		May	Aug	Nov
Migration	Catchment scale movement	3	3	3
	Movement between reaches	4	3	3
	Movement within a reach	1	3	3
Extent of the following in the reach		May	Aug	Nov
Changes in connectivity	Weirs and causeways	2	2	2
	Impoundments	1	1	1
	Physico-chemical barriers	0	0	0
	Flow modifications	1	1	1
Introduced/alien species		May	Aug	Nov
Introduced/alien species	Introduced/alien predacious species 1	MSAL	MSAL	MSAL
	Introduced/alien predacious species 2	-	-	-
	Introduced/alien predacious species 3	-	-	-
	Introduced/alien habitat modifying species 1	-	-	-
	Introduced/alien habitat modifying species 2	-	-	-
	The impact of introduced competing spp?	4	4	4
	FROC of introduced competing spp?	2.5	3	1
	The impact of introduced habitat modifying spp?	0	0	0
	FROC of habitat modifying spp?	0	0	0
**AUTOMATED FISH RESPONSE ASSESSMENT INDEX SCORE**				
FRAI (%)		4	1.2	3.9
EC: FRAI		F	F	F
**ADJUSTED FISH RESPONSE ASSESSMENT INDEX SCORE**				
FRAI (%)		48.3	46.9	48.7
EC: FRAI		D	D	D

Site Name U2KARK-USMGN **Assessor** P Dlamini
River uMngeni **Reviewed** G O'Brien

ABR	SPECIES	REFERENCE FROC	OBSERVED FROC		
			May	Aug	Nov

AMAR	ANGUILLA MARMORATA QUOY & GAIMARD 1824	5.00	-	0	0
AMOS	ANGUILLA MOSSAMBICA PETERS 1852	5.00	-	0	0
ANAT	AMPHILIUS NATALENSIS BOULENGER. 1917	5.00	-	1	0
BANO	BARBUS ANOPLUS WEBER. 1897	5.00	-	0	1
BGUR	BARBUS GURNEYI GÜNTHER. 1868	3.00	-	3	2
BNAT	BARBUS NATALENSIS CASTELNAU. 1861	5.00	-	2	2
CGAR	CLARIAS GARIEPINUS (BURCHELL. 1822)	5.00	-	0	0
OMOS	OREOCHROMIS MOSSAMBICUS (PETERS. 1852)	5.00	-	0	2
TREN	TILAPIA RENDALLI (BOULENGER. 1896)	3.00	-	0	1
TSPA	TILAPIA SPARRMANII SMITH. 1840	5.00	-	4	0
Response of species with a preference/tolerance to			May	Aug	Nov
Velocity Depth Metric	Fast-Deep		-	-1	-1
	Fast-Shallow		-	-1	-2
	Slow-Deep		-	-3	-2
	Slow-Shallow		-	-2	-1
Cover features	Overhanging veg		-	0	1
	Undercut banks		-	2	2
	Substrate		-	2	2
	Instream veg		-	1	1
	Water column		-	1	1
Response of species that are			May	Aug	Nov
Flow dependence	Intolerant to no flow		-	-2	-2
	Moderately intolerant to no flow		-	-1	-1
	Moderately tolerant to no flow		-	-3	-3
	Tolerant to no flow		-	-1	-1
Response of species that are			May	Aug	Nov
Physico-chemical conditions	Intolerant to modified physico-chemical conditions		-	0	-2
	Moderately intolerant to modified physico-chemical conditions		-	-1	0
	Moderately tolerant to modified physico-chemical conditions		-	-3	-2
	Tolerant modified to physico-chemical conditions		-	-2	-2
Response of which require			May	Aug	Nov
Migration	Catchment scale movement		-	3	2.5
	Movement between reaches		-	2	1
	Movement within a reach		-	0	1
Extent of the following in the reach			May	Aug	Nov

		May	Aug	Nov
Changes in connectivity	Weirs and causeways	-	1	1
	Impoundments	-	1	1
	Physico-chemical barriers	-	0	0
	Flow modifications	-	0	0
Introduced/alien species		May	Aug	Nov
Introduced/alien species	Introduced/alien predacious species 1	-	-	-
	Introduced/alien predacious species 2	-	-	-
	Introduced/alien predacious species 3	-	-	-
	Introduced/alien habitat modifying species 1	-	-	-
	Introduced/alien habitat modifying species 2	-	-	-
	The impact of introduced competing spp?	-	0	0
	FROC of introduced competing spp?	-	0	0
	The impact of introduced habitat modifying spp?	-	0	0
	FROC of habitat modifying spp?	-	0	0
**AUTOMATED FISH RESPONSE ASSESSMENT INDEX SCORE**				
FRAI (%)		-	34.7	29.1
EC: FRAI		-	E	E
**ADJUSTED FISH RESPONSE ASSESSMENT INDEX SCORE**				
FRAI (%)		-	70.4	69.6
EC: FRAI		-	C	C

Site Name U2MGEN-FOUNT **Assessor** P Dlamini
River uMngeni **Reviewed** G O'Brien

ABR	SPECIES	REFERENCE FROC	OBSERVED FROC		
			May	Aug	Nov
AAEN	AWAOUS AENEOFUSCUS (PETERS 1852)	5.00	0	0	-
ALAB	ANGUILLA BENGALENSIS LABIATA PETERS. 1852	3.00	0	0	-
AMAR	ANGUILLA MARMORATA QUOY & GAIMARD 1824	3.00	0	0	-
AMOS	ANGUILLA MOSSAMBICA PETERS 1852	5.00	0	0	-
ANAT	AMPHILIUS NATALENSIS BOULENGER. 1917	5.00	1	0	-
BGUR	BARBUS GURNEYI GÜNTHER. 1868	3.00	0	0	-
BNAT	BARBUS NATALENSIS CASTELNAU. 1861	5.00	2	2	-
BPAL	BARBUS PALLIDUS SMITH. 1841	1.00	0	0	-
BVIV	BARBUS VIVIPARUS WEBER. 1897	5.00	0	0	-

CGAR	CLARIAS GARIEPINUS (BURCHELL. 1822)	5.00	1	0	-
OMOS	OREOCHROMIS MOSSAMBICUS (PETERS. 1852)	5.00	0	0	-
PPHI	PSEUDOCRENILABRUS PHILANDER (WEBER. 1897)	3.00	1	0	-
TREN	TILAPIA RENDALLI (BOULENGER. 1896)	5.00	0	0	-
TSPA	TILAPIA SPARRMANII SMITH. 1840	5.00	0	2	-
Response of species with a preference/tolerance to			May	Aug	Nov
Velocity Depth Metric	Fast-Deep		-2	-2	-
	Fast-Shallow		-2	-2	-
	Slow-Deep		-4	-4	-
	Slow-Shallow		-3	-3	-
Cover features	Overhanging veg		2	2	-
	Undercut banks		2	2	-
	Substrate		2	3	-
	Instream veg		2	2	-
	Water column		2	2	-
Response of species that are			May	Aug	Nov
Flow dependence	Intolerant to no flow		-1	-3	-
	Moderately intolerant to no flow		-1	-1	-
	Moderately tolerant to no flow		-3	-3	-
	Tolerant to no flow		-2	-2	-
Response of species that are			May	Aug	Nov
Physico-chemical conditions	Intolerant to modified physico-chemical conditions		1	-3	-
	Moderately intolerant to modified physico-chemical conditions		-3	-3	-
	Moderately tolerant to modified physico-chemical conditions		-4	-2	-
	Tolerant modified to physico-chemical conditions		-2	-2	-
Response of which require			May	Aug	Nov
Migration	Catchment scale movement		1.5	2	-
	Movement between reaches		1	1	-
	Movement within a reach		1	2	-
Extent of the following in the reach			May	Aug	Nov
Changes in connectivity	Weirs and causeways		1	1	-
	Impoundments		2	2	-
	Physico-chemical barriers		0	0	-
	Flow modifications		3	3	-

Introduced/alien species		May	Aug	Nov
Introduced/alien species	Introduced/alien predacious species 1	-	-	-
	Introduced/alien predacious species 2	-	-	-
	Introduced/alien predacious species 3	-	-	-
	Introduced/alien habitat modifying species 1	-	-	-
	Introduced/alien habitat modifying species 2	-	-	-
	The impact of introduced competing spp?	0	0	-
	FROC of introduced competing spp?	0	0	-
	The impact of introduced habitat modifying spp?	0	0	-
	FROC of habitat modifying spp?	0	0	-
**AUTOMATED FISH RESPONSE ASSESSMENT INDEX SCORE**				
FRAI (%)		28.2	21.1	-
EC: FRAI		E	E/F	-
**ADJUSTED FISH RESPONSE ASSESSMENT INDEX SCORE**				
FRAI (%)		61.1	53.4	-
EC: FRAI		C/D	D	-

**Site Name**	U2DUZI-MOTOX	**Assessor**	P Dlamini
**River**	uMngeni	**Reviewed**	G O'Brien

ABR	SPECIES	REFERENCE FROC	OBSERVED FROC		
			May	Aug	Nov
AAEN	AWAOUS AENEOFUSCUS (PETERS 1852)	3.00	-	0	0
ALAB	ANGUILLA BENGALENSIS LABIATA PETERS. 1852	3.00	-	0	0
AMOS	ANGUILLA MOSSAMBICA PETERS 1852	5.00	-	0	0
ANAT	AMPHILIUS NATALENSIS BOULENGER. 1917	1.00	-	0	0
BGUR	BARBUS GURNEYI GÜNTHER. 1868	5.00	-	0	0
BNAT	BARBUS NATALENSIS CASTELNAU. 1861	5.00	-	1	0
BPAL	BARBUS PALLIDUS SMITH. 1841	1.00	-	0	0
BVIV	BARBUS VIVIPARUS WEBER. 1897	5.00	-	0	0
CGAR	CLARIAS GARIEPINUS (BURCHELL. 1822)	5.00	-	0	0
OMOS	OREOCHROMIS MOSSAMBICUS (PETERS. 1852)	5.00	-	0	0
PPHI	PSEUDOCRENILABRUS PHILANDER (WEBER. 1897)	5.00	-	0	1
TREN	TILAPIA RENDALLI (BOULENGER. 1896)	5.00	-	0	0
TSPA	TILAPIA SPARRMANII SMITH. 1840	5.00	-	2	0

Response of species with a preference/tolerance to		May	Aug	Nov
Velocity Depth Metric	Fast-Deep	-	-3	-3
	Fast-Shallow	-	-3	-4
	Slow-Deep	-	-4	-4
	Slow-Shallow	-	-3	-3
Cover features	Overhanging veg	-	2	3
	Undercut banks	-	3	3
	Substrate	-	3	4
	Instream veg	-	2	3
	Water column	-	2	2
Response of species that are		May	Aug	Nov
Flow dependence	Intolerant to no flow	-	-3	-2
	Moderately intolerant to no flow	-	-2	-2
	Moderately tolerant to no flow	-	-3	-3
	Tolerant to no flow	-	-3	-2
Response of species that are		May	Aug	Nov
Physico-chemical conditions	Intolerant to modified physico-chemical conditions	-	-4	-4
	Moderately intolerant to modified physico-chemical conditions	-	-3	-3
	Moderately tolerant to modified physico-chemical conditions	-	-3	-3
	Tolerant modified to physico-chemical conditions	-	-2	-2
Response of which require		May	Aug	Nov
Migration	Catchment scale movement	-	2	3
	Movement between reaches	-	1	3
	Movement within a reach	-	2	2
Extent of the following in the reach		May	Aug	Nov
Changes in connectivity	Weirs and causeways	-	2	2
	Impoundments	-	3	3
	Physico-chemical barriers	-	4	4
	Flow modifications	-	3	3
Introduced/alien species		May	Aug	Nov
Introduced/alien species	Introduced/alien predacious species 1	-	-	-
	Introduced/alien predacious species 2	-	-	-
	Introduced/alien predacious species 3	-	-	-
	Introduced/alien habitat modifying species 1	-	-	-

	Introduced/alien habitat modifying species 2	-	-	-
	The impact of introduced competing spp?	-	0	0
	FROC of introduced competing spp?	-	0	0
	The impact of introduced habitat modifying spp?	-	0	0
	FROC of habitat modifying spp?	-	0	0
**AUTOMATED FISH RESPONSE ASSESSMENT INDEX SCORE**				
FRAI (%)		-	17.7	10.3
EC: FRAI		-	E/F	F
**ADJUSTED FISH RESPONSE ASSESSMENT INDEX SCORE**				
FRAI (%)		-	47.6	43.4
EC: FRAI		-	D	D

**Site Name**	U2DUZI-NKANY	**Assessor**	P Dlamini
**River**	uMngeni	**Reviewed**	G O'Brien

ABR	SPECIES	REFERENCE FROC	OBSERVED FROC		
			May	Aug	Nov
AAEN	AWAOUS AENEOFUSCUS (PETERS 1852)	3.00	0	0	0
ALAB	ANGUILLA BENGALENSIS LABIATA PETERS. 1852	3.00	0	0	0
AMOS	ANGUILLA MOSSAMBICA PETERS 1852	3.00	0	0	0
ANAT	AMPHILIUS NATALENSIS BOULENGER. 1917	1.00	0	0	0
BGUR	BARBUS GURNEYI GÜNTHER. 1868	3.00	0	0	0
BNAT	BARBUS NATALENSIS CASTELNAU. 1861	3.00	3	1	1
BPAL	BARBUS PALLIDUS SMITH. 1841	1.00	0	0	0
BVIV	BARBUS VIVIPARUS WEBER. 1897	5.00	0	0	0
CGAR	CLARIAS GARIEPINUS (BURCHELL. 1822)	3.00	0	0	0
OMOS	OREOCHROMIS MOSSAMBICUS (PETERS. 1852)	3.00	0	0	0
PPHI	PSEUDOCRENILABRUS PHILANDER (WEBER. 1897)	3.00	0	0	1
TREN	TILAPIA RENDALLI (BOULENGER. 1896)	3.00	1	0	0
TSPA	TILAPIA SPARRMANII SMITH. 1840	3.00	1	1	0

Response of species with a preference/tolerance to		May	Aug	Nov
Velocity Depth Metric	Fast-Deep	-2	-2	-2
	Fast-Shallow	-2	-2	-2
	Slow-Deep	-3	-4	-4
	Slow-Shallow	-2	-3	-3

		May	Aug	Nov
Cover features	Overhanging veg	4	3	3
	Undercut banks	3	3	2
	Substrate	3	3	3
	Instream veg	1	3	4
	Water column	1	3	3
Response of species that are		May	Aug	Nov
Flow dependence	Intolerant to no flow	-1	-1	-1
	Moderately intolerant to no flow	0	-1	-1
	Moderately tolerant to no flow	-4	-4	-4
	Tolerant to no flow	-3	-3	-3
Response of species that are		May	Aug	Nov
Physico-chemical conditions	Intolerant to modified physico-chemical conditions	-2	-2	-2
	Moderately intolerant to modified physico-chemical conditions	-2	-4	-4
	Moderately tolerant to modified physico-chemical conditions	-3	-4	-4
	Tolerant modified to physico-chemical conditions	-3	-4	-3
Response of which require		May	Aug	Nov
Migration	Catchment scale movement	1.5	1	1
	Movement between reaches	1	1	1
	Movement within a reach	2.5	2.5	2.5
Extent of the following in the reach		May	Aug	Nov
Changes in connectivity	Weirs and causeways	2	2	2
	Impoundments	1	1	1
	Physico-chemical barriers	3	3	3
	Flow modifications	1	1	1
Introduced/alien species		May	Aug	Nov
Introduced/alien species	Introduced/alien predacious species 1	-	-	MSAL
	Introduced/alien predacious species 2	-	-	-
	Introduced/alien predacious species 3	-	-	-
	Introduced/alien habitat modifying species 1	-	-	-
	Introduced/alien habitat modifying species 2	-	-	-
	The impact of introduced competing spp?	0	0	4
	FROC of introduced competing spp?	0	0	1
	The impact of introduced habitat modifying spp?	0	0	0
	FROC of habitat modifying spp?	0	0	0

AUTOMATED FISH RESPONSE ASSESSMENT INDEX SCORE			
FRAI (%)	30.6	20.7	16.2
EC: FRAI	E	E/F	F

ADJUSTED FISH RESPONSE ASSESSMENT INDEX SCORE			
FRAI (%)	54.0	44.1	40.3
EC: FRAI	D	D	D/E

**Site Name**	U2MGEN-MZINY	**Assessor**	P Dlamini
**River**	uMngeni	**Reviewed**	G O'Brien

ABR	SPECIES	REFERENCE FROC	OBSERVED FROC		
			May	Aug	Nov
AAEN	AWAOUS AENEOFUSCUS (PETERS 1852)	5.00	1	1	0
ABER	ACANTHOPAGRUS BERDA (FORSSKÅL. 1775)	3.00	0	0	0
ALAB	ANGUILLA BENGALENSIS LABIATA PETERS. 1852	5.00	0	0	0
AMAR	ANGUILLA MARMORATA QUOY & GAIMARD 1824	5.00	0	0	0
AMOS	ANGUILLA MOSSAMBICA PETERS 1852	5.00	0	0	0
ANAT	AMPHILIUS NATALENSIS BOULENGER. 1917	5.00	0	0	0
BANO	BARBUS ANOPLUS WEBER. 1897	5.00	0	0	0
BGUR	BARBUS GURNEYI GÜNTHER. 1868	5.00	0	0	0
BNAT	BARBUS NATALENSIS CASTELNAU. 1861	5.00	0	0	1
BPAL	BARBUS PALLIDUS SMITH. 1841	5.00	0	0	0
BVIV	BARBUS VIVIPARUS WEBER. 1897	5.00	0	0	0
CGAR	CLARIAS GARIEPINUS (BURCHELL. 1822)	5.00	0	1	0
GAES	GILCHRISTELLA AESTUARIA (GILCHRIST. 1913)	3.00	0	0	0
GCAL	GLOSSOGOBIUS CALLIDUS SMITH. 1937	3.00	0	0	0
GGIU	GLOSSOGOBIUS GIURIS (HAMILTON-BUCHANAN. 1822)	3.00	0	0	0
LMCR	LIZA MACROLEPIS (SMITH. 1846)	3.00	0	0	0
MARG	MONODACTYLUS ARGENTEUS (LINNAEUS. 1758)	3.00	0	0	0
MBRA	MICROPHIS BRACHYURUS BLEEKER. 1853	3.00	0	0	0
MCAP	MYXUS CAPENSIS (VALENCIENNES. 1836)	3.00	0	0	0
MCEP	MUGIL CEPHALUS LINNAEUS. 1758	3.00	0	0	0
MFLU	MICROPHIS FLUVIATILIS (PETERS. 1852)	1.00	0	0	0
OMOS	OREOCHROMIS MOSSAMBICUS (PETERS. 1852)	5.00	0	1	0
PPHI	PSEUDOCRENILABRUS PHILANDER (WEBER. 1897)	5.00	2	3	1

RDEW	REDIGOBIUS DEWAALI (WEBER. 1897)	3.00	0	0	0
TREN	TILAPIA RENDALLI (BOULENGER. 1896)	5.00	1	0	0
TSPA	TILAPIA SPARRMANII SMITH. 1840	5.00	0	0	0
Response of species with a preference/tolerance to			May	Aug	Nov
Velocity Depth Metric	Fast-Deep		-3	-3	-2
	Fast-Shallow		-3	-3	-2
	Slow-Deep		-4	-3	-3
	Slow-Shallow		-4	-3	-3
Cover features	Overhanging veg		3	3	3
	Undercut banks		4	3	3
	Substrate		4	4	4
	Instream veg		4	4	4
	Water column		4	4	3
Response of species that are			May	Aug	Nov
Flow dependence	Intolerant to no flow		-2	-2	-2
	Moderately intolerant to no flow		-3	-3	-2
	Moderately tolerant to no flow		-4	-4	-4
	Tolerant to no flow		-2	-2	-4
Response of species that are			May	Aug	Nov
Physico-chemical conditions	Intolerant to modified physico-chemical conditions		-2	-2	-2
	Moderately intolerant to modified physico-chemical conditions		-3	-3	-3
	Moderately tolerant to modified physico-chemical conditions		-4	-4	-2.5
	Tolerant modified to physico-chemical conditions		-3	-3	-3
Response of which require			May	Aug	Nov
Migration	Catchment scale movement		2.5	2.5	3
	Movement between reaches		2.5	2	3
	Movement within a reach		2	2	3
Extent of the following in the reach			May	Aug	Nov
Changes in connectivity	Weirs and causeways		3	3	3
	Impoundments		3	3	3
	Physico-chemical barriers		1	1	1
	Flow modifications		3	3	3
Introduced/alien species			May	Aug	Nov
	Introduced/alien predacious species 1		MSAL		

	Introduced/alien predacious species 2	-		
	Introduced/alien predacious species 3	-		
	Introduced/alien habitat modifying species 1	-		
Introduced/alien species	Introduced/alien habitat modifying species 2	-		
	The impact of introduced competing spp?	4		
	FROC of introduced competing spp?	1		
	The impact of introduced habitat modifying spp?	0		
	FROC of habitat modifying spp?	0		
**AUTOMATED FISH RESPONSE ASSESSMENT INDEX SCORE**				
FRAI (%)		12.3	17.2	14.3
EC: FRAI		F	F	F
**ADJUSTED FISH RESPONSE ASSESSMENT INDEX SCORE**				
FRAI (%)		37.2	44.1	44.0
EC: FRAI		E	D	D
